# Picomolar, selective, and subtype-specific small-molecule inhibition of TRPC1/4/5 channels

**DOI:** 10.1074/jbc.M116.773556

**Published:** 2017-03-21

**Authors:** Hussein N. Rubaiy, Melanie J. Ludlow, Matthias Henrot, Hannah J. Gaunt, Katarina Miteva, Sin Ying Cheung, Yasuyuki Tanahashi, Nurasyikin Hamzah, Katie E. Musialowski, Nicola M. Blythe, Hollie L. Appleby, Marc A. Bailey, Lynn McKeown, Roger Taylor, Richard Foster, Herbert Waldmann, Peter Nussbaumer, Mathias Christmann, Robin S. Bon, Katsuhiko Muraki, David J. Beech

**Affiliations:** From the Schools of ‡ Medicine and; ‖ Chemistry, University of Leeds, Leeds LS2 9JT, United Kingdom,; the § Institute of Chemistry and Biochemistry, Freie Universität Berlin, Takustrasse 3, 14195 Berlin, Germany,; the ¶ Faculty of Life Sciences, Kyoto Sangyo University, Kyoto 603-8555, Japan,; the ** Max-Planck-Institut für Molekulare Physiologie, Otto-Hahn-Strasse 11, 44227 Dortmund, Germany,; ‡‡ Lead Discovery Center GmbH, Otto-Hahn-Strasse 15, D-44227 Dortmund, Germany, and; the §§ School of Pharmacy, Aichi-Gakuin University, 1-100 Kusumoto, Chikusa, Nagoya 464-8650, Japan

**Keywords:** calcium, calcium release-activated calcium channel protein 1 (ORAI1), ion channel, pharmacology, transient receptor potential channels (TRP channels), englerin, heteromeric channel, non-selective cationic channel, store-operated calcium entry, transient receptor potential canonical

## Abstract

The concentration of free cytosolic Ca^2+^ and the voltage across the plasma membrane are major determinants of cell function. Ca^2+^-permeable non-selective cationic channels are known to regulate these parameters, but understanding of these channels remains inadequate. Here we focus on transient receptor potential canonical 4 and 5 proteins (TRPC4 and TRPC5), which assemble as homomers or heteromerize with TRPC1 to form Ca^2+^-permeable non-selective cationic channels in many mammalian cell types. Multiple roles have been suggested, including in epilepsy, innate fear, pain, and cardiac remodeling, but limitations in tools to probe these channels have restricted progress. A key question is whether we can overcome these limitations and develop tools that are high-quality, reliable, easy to use, and readily accessible for all investigators. Here, through chemical synthesis and studies of native and overexpressed channels by Ca^2+^ and patch-clamp assays, we describe compound 31, a remarkable small-molecule inhibitor of TRPC1/4/5 channels. Its potency ranged from 9 to 1300 pm, depending on the TRPC1/4/5 subtype and activation mechanism. Other channel types investigated were unaffected, including TRPC3, TRPC6, TRPV1, TRPV4, TRPA1, TRPM2, TRPM8, and store-operated Ca^2+^ entry mediated by Orai1. These findings suggest identification of an important experimental tool compound, which has much higher potency for inhibiting TRPC1/4/5 channels than previously reported agents, impressive specificity, and graded subtype selectivity within the TRPC1/4/5 channel family. The compound should greatly facilitate future studies of these ion channels. We suggest naming this TRPC1/4/5-inhibitory compound Pico145.

## Introduction

Transient receptor potential canonical (TRPC)^4^ proteins assemble as multimers around a central ion pore to enable Ca^2+^ and Na^+^ entry into mammalian cells (1, 2). The multimers may be repeats of the same TRPC protein (homomers) or mixtures of different TRPC proteins (heteromers). A group of TRPC proteins that are thought to cluster together consists of TRPC1, TRPC4, and TRPC5 (TRPC1/4/5). TRPC1 was first reported in 1995, and since then it has been found to be broadly expressed (1–3). Among the TRPC proteins, it is unusual in failing to form ion channels or forming them poorly when overexpressed alone in cell lines (2, 4–6). Overexpressed TRPC1, by contrast, readily forms functional heteromers with TRPC4 and TRPC5 (5, 7, 8). TRPC4 and TRPC5 are also capable of forming functional homomers without TRPC1, but these might be relatively uncommon in physiology because TRPC1 is so widely expressed. The specific functions and mechanisms of the TRPC1/4/5 channels and the relative importance of homomers and heteromers remain poorly understood. One of the many challenges has been to determine their relevance in store-operated Ca^2+^ entry, a type of Ca^2+^ entry seen in most cell types when the intracellular Ca^2+^ stores are depleted (9).

A key restriction on TRPC1/4/5 studies has been the lack of pharmacological tools with which to specifically activate or inhibit the channels (10). An important recent breakthrough was the discovery of (−)-englerin A (EA), a highly efficacious, potent, and apparently specific activator of TRPC1/4/5 channels (homomers and heteromers) (8, 11, 12). For investigations of the physiological significance and translational potential of the channels, however, there is a pressing need for potent and specific inhibitors. Probably, the most characterized inhibitor reported so far is the TRPC4/5 inhibitor ML204 (13). It inhibited TRPC4 with an IC_50_ of about 1 μm and caused about 65% inhibition of TRPC5 at 10 μm (13). This is not particularly potent, and, as we show in the current study, there is a weaker effect on TRPC1-containing channels.

Despite the limitations of the TRPC1/4/5 tools, there is compelling evidence for important roles of the channels, notably in animal models of human pathophysiology and clinical samples. The channels are suggested to have roles in epilepsy, innate fear, pain, rheumatoid arthritis, and adverse cardiac remodeling, for example (10, 14–21). TRPC4 and TRPC5 knock-out mice exhibited reduced innate fear and reduced anxiety (16, 17). TRPC5 has been implicated in the regulation of adiponectin secretion (22), kidney barrier function (23), and baroreceptor control of blood pressure (24). TRPC4 knock-out mice and mice treated with ML204 showed less body licking and abdominal retractions in response to mustard oil (18). TRPC4 and TRPC5 contributed to maintenance of pain hypersensitivity and neuropathy (25). TRPC1 was up-regulated and had a positive role in neointimal hyperplasia of the human saphenous vein, where it may function in partnership with TRPC5 (4, 26). In vascular smooth muscle cells from the human saphenous vein, TRPC5-dependent channels were activated by sphingosine 1-phosphate, and this drove cell migration. Other studies point to roles in aspects of cancer: drug resistance and its transmission via extracellular vesicles, tumor vascularization, and cancer cell death evoked by EA (27).

Compound 31 (C31) was specified in an international patent application, where it was suggested to be a pharmacologically effective inhibitor of TRPC5 channels (28). We initially synthesized this compound to independently confirm its effectiveness as a TRPC5 inhibitor. We found that it is an inhibitor of TRPC5 and so progressed to determining whether it has effects on other members of this protein family. We found remarkable effects, particularly on TRPC4 and importantly also its heteromer with TRPC1.

## Results

The chemical structure of C31 is shown in supplemental Fig. S1. C31 was synthesized according to three different synthetic routes, all providing analytically pure material according to NMR and LC-MS analysis (see supplemental Figs. S2–S4 and Schemes S1–S3).

### C31 is a nanomolar inhibitor of TRPC5

**Figure 1.**
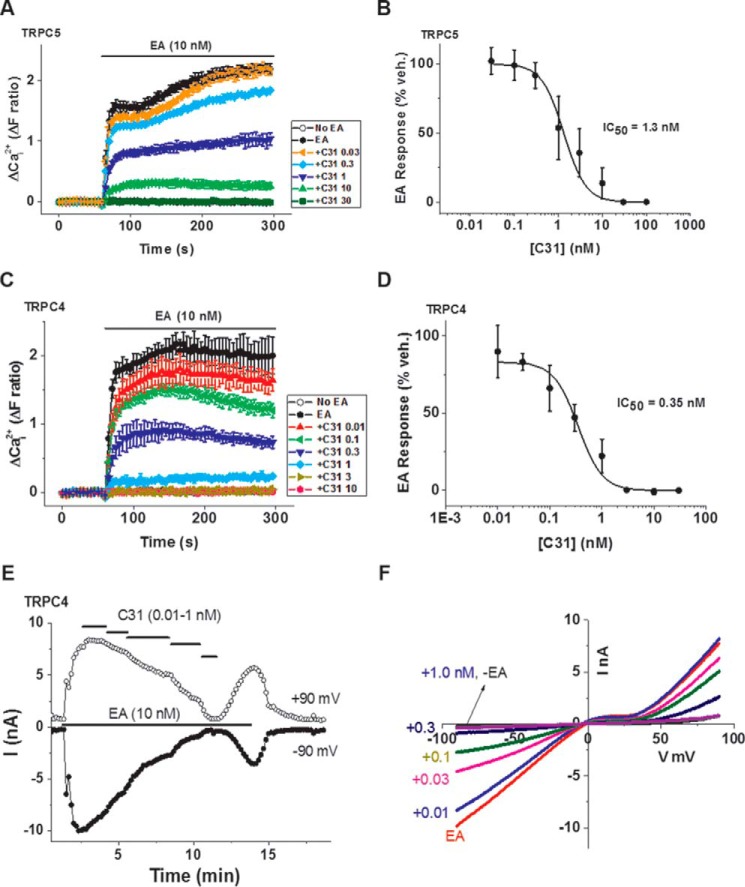
**C31 inhibits TRPC5 and TRPC4 homomeric channels.** *A*, example Ca^2+^ measurement data from a single 96-well plate showing concentration-dependent inhibition of EA (10 nm)-evoked Ca^2+^ entry by 0.03, 0.3, 1, 10, and 30 nm C31 in HEK 293 Tet^+^ cells expressing human TRPC5 (*N* = 4/data point). *B*, summary data for experiments of the type shown in *A* plotted as a percentage of the response to EA in the vehicle control for C31 (*n* = 6 independent experiments). The *fitted curve* is the Hill equation with IC_50_ = 1.32 nm (*n*/*N* = 6/24). *C*, example Ca^2+^ measurement data from a single 96-well plate showing concentration-dependent inhibition of EA (10 nm)-evoked Ca^2+^ entry by 0.01, 0.1, 0.3, 1, 3, and 10 nm C31 in HEK 293 Tet^+^ cells expressing human TRPC4 (*N* = 4/data point). *D*, summary data for experiments of the type shown in *C* plotted as a percentage of the response to EA in the vehicle control for C31 (*n* = 6 independent experiments). The *fitted curve* is the Hill equation with IC_50_ = 0.349 nm (*n*/*N* = 6/24). *E*, example whole-cell patch-clamp data from a TRPC4-expressing HEK 293 cell showing current sampled at −90 and +90 mV during ramp changes in voltages. EA and C31 were bath-applied at the concentrations indicated (C31, 0.01, 0.03, 0.1, 0.3, and 1 nm). *F*, example IVs from the experiment in *E*. The *numbers* to the *left* specify C31 concentrations in nm. −*EA*, indicates current sampled before EA was applied. *Error bars*, S.D.

To observe the functional effects of C31 on TRPC5, we used human embryonic kidney (HEK) 293 cells containing stably incorporated tetracycline-inducible expression of exogenous human TRPC5. The cells were in 96-well plates and loaded with the intracellular Ca^2+^ indicator fura-2 to detect Ca^2+^ entry via activated TRPC5 channels. Application of 10 nm EA caused robust activation of the channels (Fig. 1*A*), similar to previous observations (8). Cells preincubated and maintained in the presence of 30 nm C31 failed to show responses to EA (Fig. 1*A*). Investigation of other concentrations of C31 revealed the concentration for 50% inhibition (IC_50_) to be 1.3 nm (Fig. 1, *A* and *B*). The data suggest that C31 was a nanomolar inhibitor of EA-activated TRPC5 channels.

### C31 is more potent against TRPC4

We next investigated TRPC4 using a similar approach to that for TRPC5. EA is also an agonist at TRPC4 channels with a concentration for 50% effect (EC_50_) similar to that seen with TRPC5 (11.2 nm; *cf.* 7.6 nm) (8). It was therefore used again at 10 nm to stimulate the channels. C31 inhibited TRPC4, but in this case, 3 nm C31 was sufficient for complete inhibition, and the IC_50_ was 0.349 nm (Fig. 1, *C* and *D*). The data suggest that C31 inhibited TRPC4 as well as TRPC5 and that it was more potent against TRPC4.

### C31 may act directly to inhibit the channels

**Figure 2.**
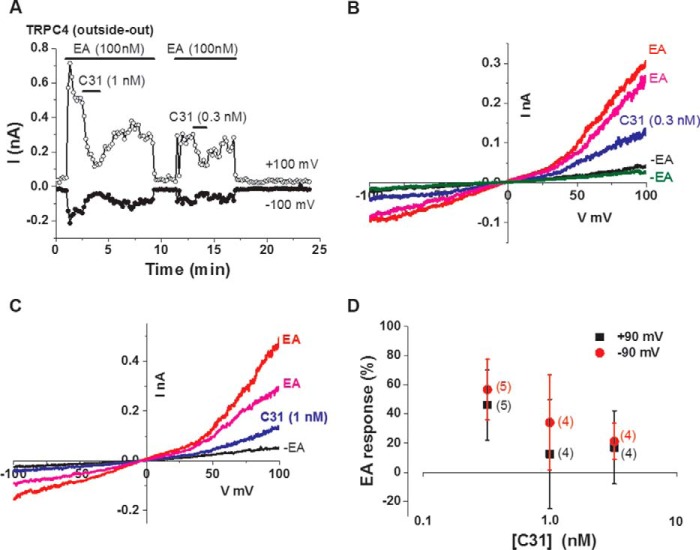
**C31 inhibits TRPC4 channel currents in outside-out patches.** *A*, example outside-out patch-clamp data from a TRPC4-expressing HEK 293 cell showing current sampled at −100 and +100 mV during ramp changes in voltages. EA and C31 were bath-applied at the concentrations indicated. *B*, example IVs from the experiment in *A* during the second application of EA. The EA response before C31 is shown in *red* and after washout of C31 in *pink*. Before and after washout of EA are indicated by *EA. C*, example outside-out patch-clamp data from a TRPC4-expressing HEK 293 cell displaying IVs during the first application of EA as shown in *A*. The EA response before C31 is shown in *red* and after washout of C31 in *pink*. Current before EA is indicated by −*EA. D*, mean concentration-response data for experiments for the type shown in *A*, *B*, and *C*. The numbers of independent experiments are indicated in *parentheses. Error bars*, S.D.

Because the interpretation of Ca^2+^ measurement data can be complicated by indirect effects on cellular Ca^2+^ handling and membrane potential, we further investigated the effect of C31 by patch recording. First we used whole-cell recording, in which current through TRPC4 channels was measured across a range of voltages. C31 strongly and concentration-dependently inhibited the TRPC4-mediated ionic current, and its effect was at least partially reversible on washout (Fig. 1*E*). The ionic current showed the characteristic seatlike current-voltage relationship (IV) of TRPC4 channels, and the inhibition was similar at negative and positive voltages (Fig. 1*F*). Second, we used excised outside-out membrane patch recording in which the patch was detached from the cell, and its ionic environment was strongly controlled. We used 100 nm EA to maximize the size and clarity of the ionic current against which the effect of C31 could be tested (Fig. 2*A*). C31 inhibited the TRPC4-mediated ionic current in a concentration-dependent manner, and its effect was reversible on washout (Fig. 2, *A–D*). The data suggest that C31 inhibited the channels independently of cellular constituents, other Ca^2+^-handling mechanisms, or membrane potential. The data are suggestive of C31 acting directly on the channels.

### Heteromers of TRPC1 with TRPC4/5 are pharmacologically distinct

**Figure 3.**
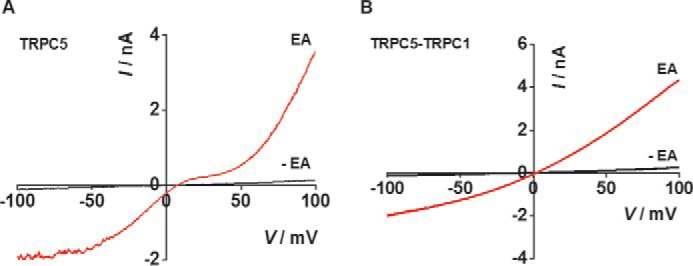
**TRPC5 homomer and TRPC5-TRPC1 concatemer have distinct IVs.** Typical whole-cell IVs are shown for TRPC5 expressed alone (A) and the TRPC5-TRPC1 concatemer before (−*EA*, *black*) and after bath application of 100 nm EA (*red*) (*B*). The IVs were generated using voltage ramps from +100 to −100 mV delivered every 10 s.

**Figure 4.**
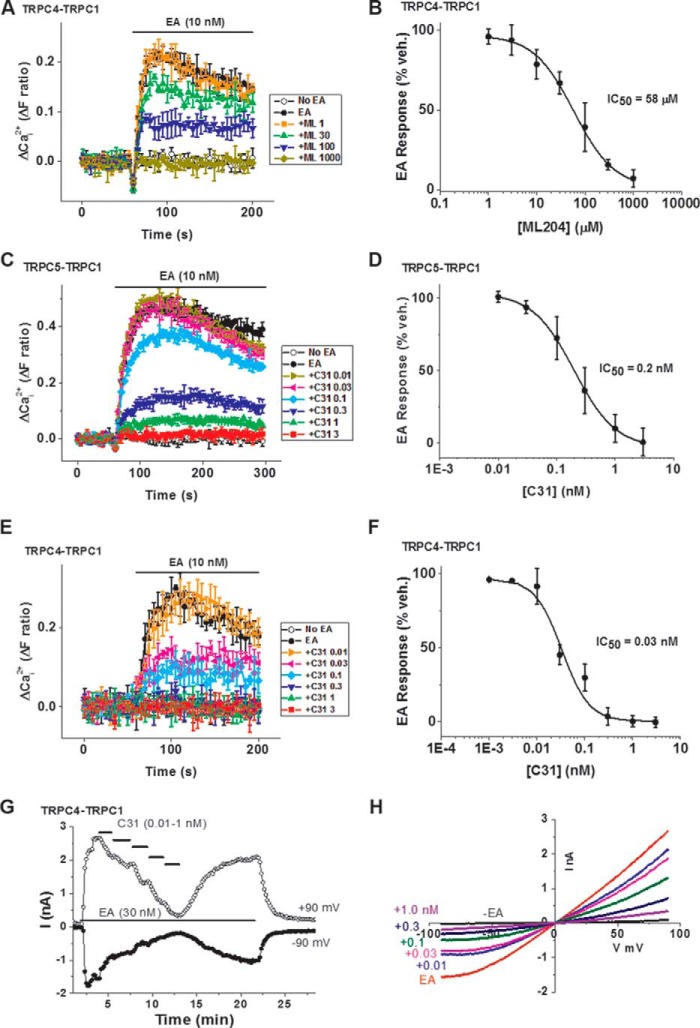
**C31 inhibits heteromeric channels.** *A*, example Ca^2+^ measurement data from a single 96-well plate showing concentration-dependent inhibition of EA (10 nm)-evoked Ca^2+^ entry by 1, 30, 100, and 1000 μm ML204 in HEK 293 Tet^+^ cells expressing human TRPC4-TRPC1 concatemer (*N* = 4/data point). *B*, summary data for experiments of the type shown in *A* plotted as a percentage of the response to EA in the vehicle control for ML204 (*n* = 6 independent experiments). The *fitted curve* is the Hill equation with IC_50_ = 58 μm (*n*/*N* = 6/24). *C*, example Ca^2+^ measurement data from a single 96-well plate showing concentration-dependent inhibition of EA (10 nm)-evoked Ca^2+^ entry by 0.01, 0.03, 0.1, 0.3, 1, and 3 nm C31 in HEK 293 Tet^+^ cells expressing human TRPC5-TRPC1 concatemer (*N* = 3/data point). *D*, summary data for experiments of the type shown in *C* plotted as a percentage of the response to EA in the vehicle control for C31 (*n* = 8 independent experiments). The *fitted curve* is the Hill equation with IC_50_ = 0.2 nm (*n*/*N* = 8/24). *E* and *F*, similar to *C* and *D* but using HEK 293 Tet^+^ cells expressing the human TRPC4-TRPC1 concatemer and resulting in a Hill equation with IC_50_ = 0.033 nm (*n*/*N* = 6/24). *G*, example whole-cell current recording from a HEK 293 Tet^+^ cell expressing the TRPC4-TRPC1 concatemer showing current sampled at −90 and +90 mV during ramp changes in voltages. Bath-applied as indicated were 30 nm EA and 0.01, 0.03, 0.1, 0.3, and 1 nm C31. *H*, example IVs for the experiment in *G. Numbers* to the *left* indicate the addition of C31 with concentrations in nm. *Error bars*, S.D.

Because of the importance of heteromers containing TRPC1, we generated concatemers of TRPC4 with TRPC1 and TRPC5 with TRPC1, stably incorporating them in HEK 293 cells for tetracycline-inducible expression. The concatemers formed functional channels with fingerprint single-curve IVs, which contrasted with the seatlike IVs of TRPC4 and TRPC5 (*e.g.* Figs. 1*F* and 3 (*A* and *B*)). The pharmacology of the concatemers was first investigated using a fura-2 Ca^2+^ indicator. The signals were small relative to those of homomers because TRPC1 suppresses Ca^2+^ permeability (6). ML204, the previously reported low micromolar inhibitor of TRPC4, was active against TRPC4-TRPC1 concatemer only at relatively high concentrations (IC_50_ = 58 μm) (Fig. 4, *A* and *B*). The data suggest relative resistance of TRPC4-TRPC1 heteromer to ML204.

### Heteromers with TRPC1 are potently inhibited by C31

In contrast to ML204, C31 was potent against Ca^2+^ entry through heteromers (Fig. 4, *C–F*). As little as 3 nm C31 was sufficient to prevent Ca^2+^ entry through TRPC5-TRPC1 (IC_50_ =0.199 nm) (Fig. 4, *C* and *D*). Even less C31 (0.3 nm) abolished Ca^2+^ entry through TRPC4-TRPC1 (IC_50_ = 0.033 nm) (Fig. 4, *E* and *F*). TRPC4-TRPC1 channels were also investigated by whole-cell patch-clamp recording, where C31 caused potent concentration-dependent inhibition, and its effect was largely reversible on washout (Fig. 4, *G* and *H*). The data suggest that C31 is a potent inhibitor of Ca^2+^ entry through heteromers.

### Potency of C31 is reduced by elevated EA concentration

**Figure 5.**
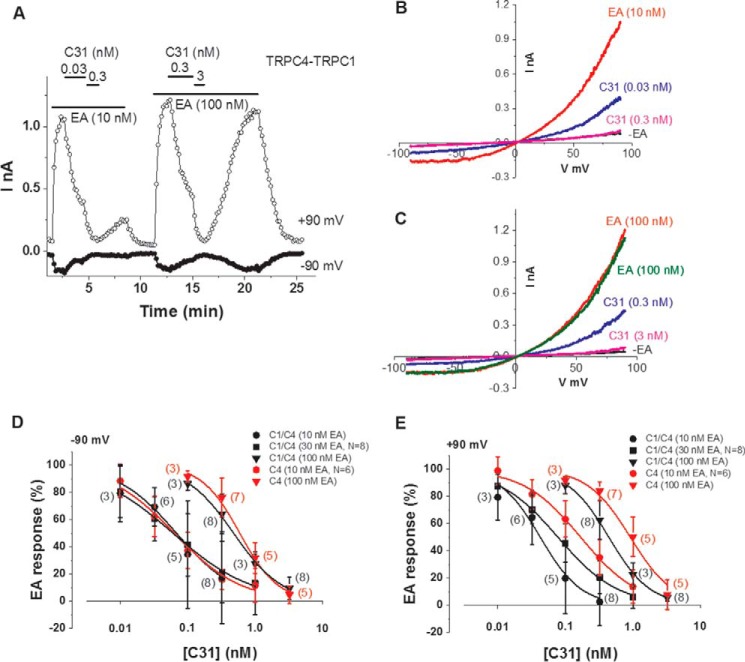
**EA negatively modulates C31 potency.** *A*, example whole-cell patch-clamp data from a TRPC4-TRPC1-expressing HEK 293 cell showing current sampled at −90 and +90 mV during ramp changes in voltages. EA and C31 were bath-applied at the concentrations indicated. *B* and *C*, example IVs from the experiment in *A* for 10 nm EA (*B*) and 100 nm EA (*C*) applications. C31 was in addition to the EA. *D* and *E*, mean concentration-response data for current amplitudes sampled at −90 mV (*D*) and +90 mV (*E*) during ramp changes in voltages in TRPC4-TRPC1 (*C4-C1*, *black symbols*) or TRPC4 (*C4*, *red symbols*)-expressing HEK 293 cells studied at the indicated EA concentrations. The numbers of independent experiments are indicated in *parentheses*. The *fitted curves* are Hill equations that indicated IC_50_ values as follows. *D*, for TRPC4-TRPC1, the IC_50_ values were 0.068 nm (10 nm EA), 0.061 nm (30 nm EA), and 0.481 nm (100 nm EA); for TRPC4, the IC_50_ values were 0.063 nm (10 nm EA) and 0.593 nm (100 nm EA). *E*, for TRPC4-TRPC1, the IC_50_ values were 0.042 nm (10 nm EA), 0.078 nm (30 nm EA), and 0.441 nm (100 nm EA); for TRPC4, the IC_50_ values were 0.169 nm (10 nm EA) and 0.916 nm (100 nm EA). *Error bars*, S.D.

EA and C31 are chemically distinct, but both are clearly potent modulators of the channels, so we considered the possibility of an interaction between their effects. We used whole-cell patch-clamp recording to compare the concentration dependence of C31 blockade in the presence of 10 and 100 nm EA. Example data for TRPC4-TRPC1 are shown (Fig. 5, *A–C*). C31 (0.3 nm) caused almost complete blockade in 10 nm EA but only partial blockade in 100 nm EA (Fig. 5, *A–C*). The recovery from C31 blockade was less good in 10 nm EA compared with 100 nm EA (Fig. 5*A*), the explanation for which was unclear. The data suggest that EA reduced the potency of C31 at TRPC4-TRPC1 channels.

Concentration-response curves were constructed for C31 in the presence of 10, 30, and 100 nm EA for TRPC4-TRPC1 channels and in the presence of 10 and 100 nm EA for TRPC4 channels (Fig. 5, *D* and *E*). The effects were measured at −90 and +90 mV. At −90 mV, the potency of C31 was similar for 10 and 30 nm EA acting at TRPC4-TRPC1 channels and 10 nm EA acting at TRPC4 channels (Fig. 5*D*). But when 100 nm EA activated TRPC4-TRPC1 or TRPC4 channels, the potency of C31 was substantially less (Fig. 5*D*). At +90 mV, the potency of C31 was best for 10 nm EA at TRPC4-TRPC1 channels and less good for 30 nm EA (Fig. 5*E*). The potency was less for 10 nm EA at TRPC4 channels (Fig. 5*E*). Again, when 100 nm EA activated TRPC4-TRPC1 or TRPC4 channels, the potency of C31 was less good (Fig. 5*E*).

The data suggest that the potency of C31 was regulated by EA concentration, becoming less with increased EA concentration. The data also suggest mild voltage dependence in the potency of C31 and reinforce the concept of graded subtype specificity.

### C31 potently inhibits channels activated by sphingosine 1-phosphate (S1P)

**Figure 6.**
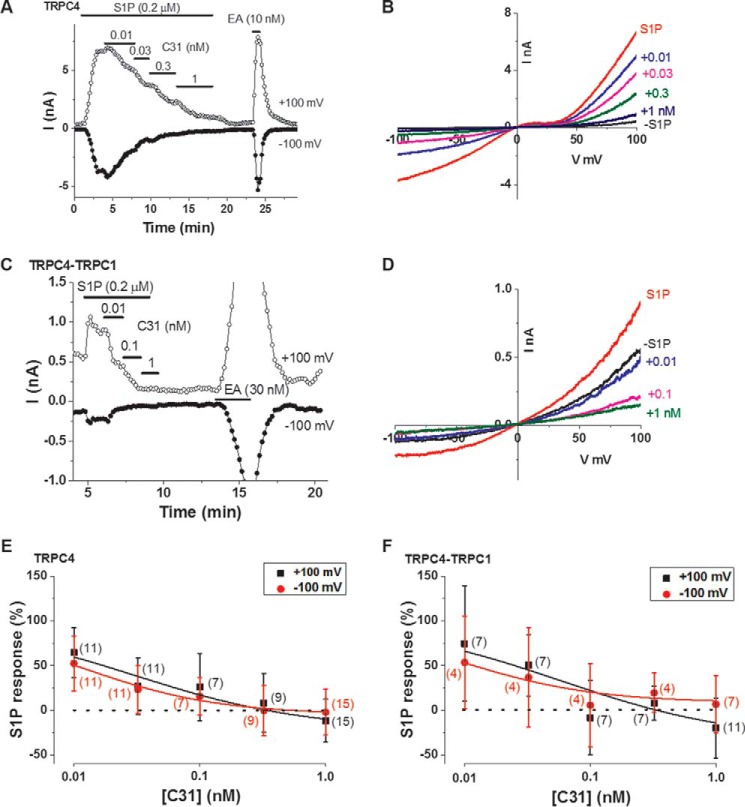
**C31 inhibits channels activated by a physiological agonist.** *A*, example whole-cell patch-clamp data from a TRPC4-expressing HEK 293 cell showing current sampled at −100 and +100 mV during ramp changes in voltages. S1P, C31, and EA were bath-applied at the concentrations indicated. *B*, example IVs from the experiment in *A* in the absence (−*S1P*) and presence of S1P plus C31 at the indicated concentrations (*e.g.* +*0.01* represents 0.01 nm C31). *C* and *D*, as for *A* and *B* but for a TRPC4-TRPC1-expressing HEK 293 cell. *E* and *F*, mean concentration-response data for current amplitudes sampled at −100 mV (*red*) and +100 mV (*black*) during ramp changes in voltages in TRPC4 (*E*)- and TRPC4-TRPC1 (*F*)-expressing HEK 293 cells studied as illustrated in *A–D*. The numbers of independent experiments are indicated in *parentheses*. The *fitted curves* are Hill equations that suggested approximate IC_50_ values as follows: 0.012 nm (−100 mV) and 0.030 nm (+100 mV) (*E*); 0.009 nm (−100 mV) and 0.059 nm (+100 mV) (*F*). *Error bars*, S.D.

The impact of EA concentration led us to ask whether the action of C31 required EA as the agonist. We therefore activated channels instead with S1P, a physiological substance that acts via a G protein signaling pathway (4, 29). We again focused on TRPC4 and TRPC4-TRPC1 channels. The S1P responses tended to be smaller and less robust than EA responses, especially for TRPC4-TRPC1 channels (Fig. 6, *A–D*). C31 was a potent and concentration-dependent inhibitor of both channel types (Fig. 6, *A–D*). S1P-activated TRPC4 and TRPC4-TRPC1 channels showed similar sensitivities to C31. For TRPC4 channels, the IC_50_ values at −100 and +100 mV were estimated to be 0.012 and 0.030 nm, respectively (Fig. 6*E*). For TRPC4-TRPC1 channels, the IC_50_ values at −100 and +100 mV were estimated to be 0.009 and 0.059 nm, respectively (Fig. 6*F*).

**Figure 7.**
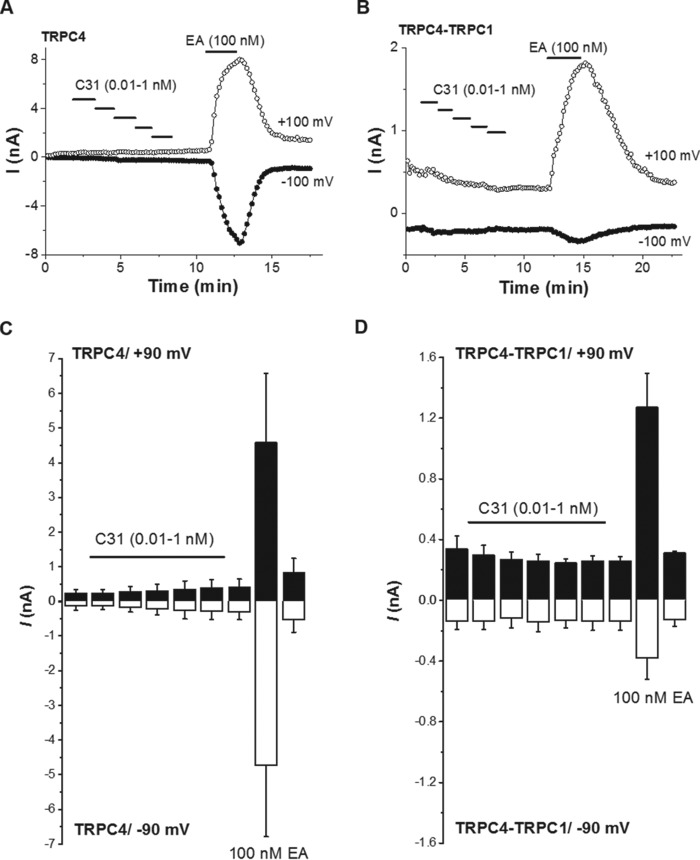
**C31 has little or no effect in the absence of exogenous channel agonists.** *A*, example whole-cell patch-clamp data from a TRPC4-expressing HEK 293 cell showing current sampled at −100 and +100 mV during ramp changes in voltage. C31 and EA were bath-applied at the concentrations indicated. *B*, example whole-cell patch-clamp data from a TRPC4-TRPC1-expressing HEK 293 cell showing current sampled at −100 and +100 mV during ramp changes in voltages. C31 and EA were bath-applied at the concentrations indicated. *A* and *B*, C31 was applied at 0.01, 0.03, 0.1, 0.3, and 1 nm. *C* and *D*, mean absolute current amplitude data for the types of experiment illustrated in *A* and *B* (*n* = 3 each). *Error bars*, S.D.

In studies of TRPC4-TRPC1 channels, we observed basal current before application of S1P and observed that C31 sometimes caused the current amplitude to decrease below the pre-S1P amplitude (Fig. 6, *C–F*). We suspected that the basal current reflected constitutive activity of TRPC4-TRPC1 channels or activity relating to endogenous (background) channels. In the absence of S1P, C31 had no effect on basal ionic current in TRPC4-expressing cells and no effect or a small inhibitory effect on basal current in TRPC4-TRPC1-expressing cells (Fig. 7).

**Figure 8.**
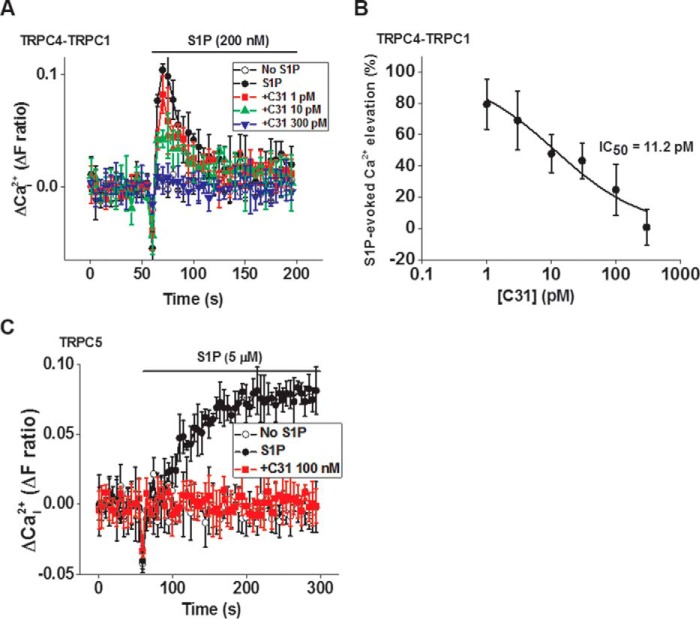
**C31 inhibits Ca^2+^ entry evoked by S1P.** *A*, example Ca^2+^ measurement data from a single 96-well plate showing concentration-dependent inhibition of S1P (200 nm)-evoked Ca^2+^ entry by 1, 10, and 300 pm C31 in HEK 293 Tet^+^ cells expressing TRPC4-TRPC1 channels (*N* = 4/data point). *B*, summary data for experiments of the type shown in *A* plotted as a percentage of the response to S1P in the vehicle control for C31. The *fitted curve* is the Hill equation with IC_50_ 11.2 pm. *C*, example Ca^2+^ measurement data from a single 96-well plate showing inhibition of S1P (5 μm)-evoked Ca^2+^ entry by 100 nm C31 in HEK 293 Tet^+^ cells expressing TRPC5 channels (*N* = 4/data point). The Ca^2+^ entry evoked by S1P was abolished by 100 nm C31 in all experiments (*n*/*N* = 6/24 each). *Error bars*, S.D.

S1P-evoked Ca^2+^ entry through TRPC4-TRPC1 channels was also potently inhibited by C31 (IC_50_ = 0.011 nm) (Fig. 8, *A* and *B*). S1P-evoked TRPC5-mediated Ca^2+^ entry was also inhibited by C31 (Fig. 8*C*).

The data suggest that the action of C31 did not depend on EA and that it had potent effects against channel activity evoked by the physiological agonist, S1P.

### C31 has more complex effects on channels activated by gadolinium ion (Gd^3+^)

**Figure 9.**
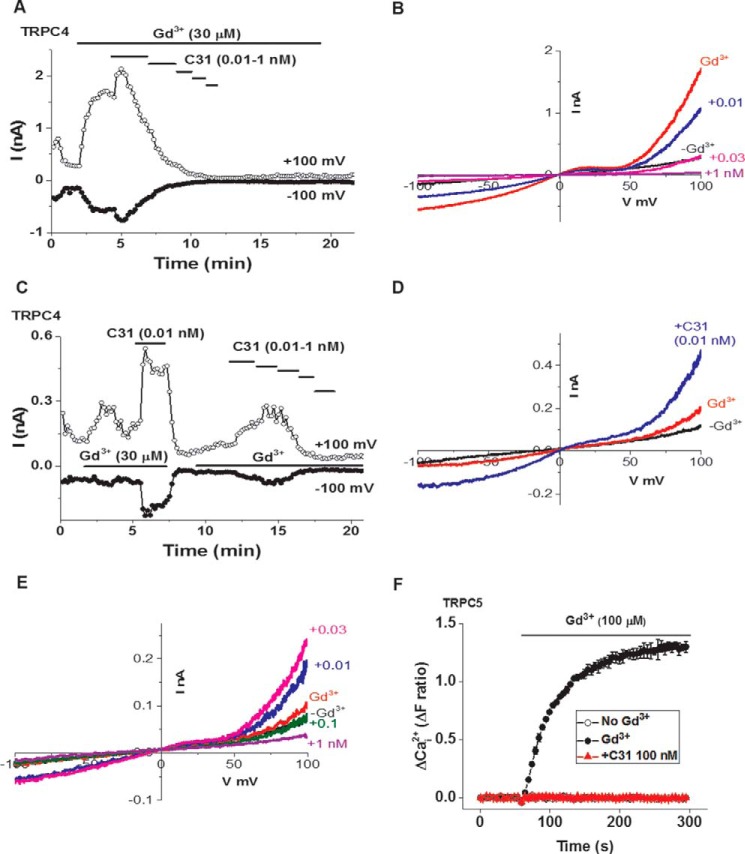
**Potentiation and inhibition of channel activity in the presence of Gd^3+^.** *A*, example whole-cell patch-clamp data from a TRPC4-expressing HEK 293 cell showing current sampled at −100 and +100 mV during ramp changes in voltages. Gd^3+^ and C31 were bath-applied at the concentrations indicated (C31, 0.01, 0.03, 0.1, 0.3, and 1 nm). *B*, example IVs from the experiment in *A* in the absence (−Gd^3+^) and presence of Gd^3+^ plus C31 at the indicated concentrations (*e.g.* +*0.01* represents 0.01 nm C31). *C*, example whole-cell patch-clamp data from a TRPC4-expressing HEK 293 cell showing current sampled at −100 and +100 mV during ramp changes in voltages. Gd^3+^ and C31 were bath-applied at the concentrations indicated (C31, 0.01, 0.03, 0.1, 0.3, and 1 nm). *D*, for the first application of Gd^3+^ shown in *C*, example IVs in the absence (−*Gd*^3+^) and presence of Gd^3+^ plus 0.01 nm C31. *E*, for the second application of Gd^3+^ shown in *C*, example IVs in the absence (−*Gd*^3+^) and presence of Gd^3+^ plus C31 at the indicated concentrations (*e.g.* +*0.01* represents 0.01 nm C31). *A–E*, the examples shown were selected as exemplary from four independent recordings. *F*, example Ca^2+^ measurement data from a single 96-well plate showing inhibition of Gd^3+^ (100 μm)-evoked Ca^2+^ entry by 100 nm C31 in HEK 293 Tet^+^ cells expressing TRPC5 channels (*N* = 4/data point). The Ca^2+^ entry evoked by S1P was abolished by 100 nm C31 in all experiments (*n*/*N* = 6/24 each). *Error bars*, S.D.

TRPC4 and TRPC5 channels are also stimulated by ions, such as Gd^3+^, and these effects might reflect a role of the channels in detecting toxic metal ions (30, 31). The stimulatory effect was not as robust as that of EA, but TRPC4-mediated currents were detected in response to Gd^3+^ (Fig. 9). Unexpectedly, low concentrations of C31 were agonistic in the presence of Gd^3+^, although higher concentrations were inhibitory (Fig. 9, *A–E*). Gd^3+^ did not activate TRPC4-TRPC1 channels (*n* = 4). C31 (100 nm) was tested against TRPC5-mediated Ca^2+^ entry and was inhibitory (Fig. 9*F*). The data suggest that Gd^3+^ enabled an agonist effect of low concentrations of C31 but that higher concentrations of C31 were inhibitory.

### C31 has specificity for TRPC1/4/5 channels

**Figure 10.**
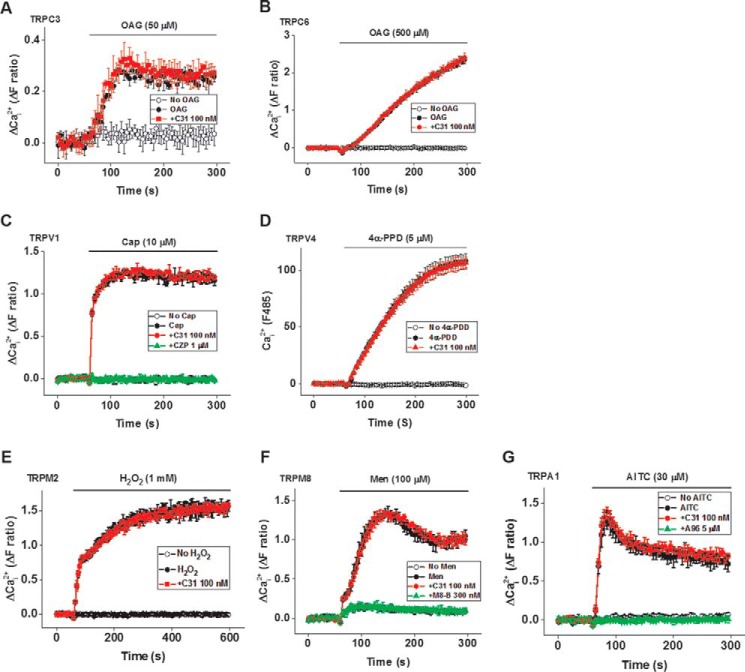
**C31 has no effect on seven other types of Ca^2+^-permeable channels.** *A* and *B*, example Ca^2+^ measurement data from a single 96-well plate showing no effect of 100 nm C31 on OAG (50 and 500 μm)-evoked Ca^2+^ entry into HEK 293 Tet^+^ cells expressing human TRPC3 (*N* = 6/data point, *n*/*N* = 6/36) and TRPC6 (transient transfected HEK 293, *N* = 3/data point, *n*/*N* = 4/12), respectively. *C*, example Ca^2+^ measurement data from a single 96-well plate showing no effect of 100 nm C31 on capsaicin (*Cap*; 10 μm)-evoked Ca^2+^ entry and full inhibition by capsazepine (*CZP*; 1 μm), in transient transfected HEK 293 cells expressing human TRPV1 (*N* = 3/data point, *n*/*N* = 4/12). *D*, example Ca^2+^ measurement data from a single 96-well plate showing no effect of 100 nm C31 on 4α-phorbol-12,13-didecanoate (*4*α*PDD*; 5 μm)-evoked Ca^2+^ entry into CHO cells stably expressing human TRPV4 (*N* = 3/data point, *n*/*N* = 6/18). *E*, example Ca^2+^ measurement data from a single 96-well plate showing no effect of 100 nm C31 on hydrogen peroxide (H_2_O_2_; 1 mm)-evoked Ca^2+^ entry into HEK 293 Tet^+^ cells expressing human TRPM2 (*N* = 6/data point, *n*/*N* = 6/36). *F*, example Ca^2+^ measurement data from a single 96-well plate showing no effect of 100 nm C31 on menthol (*Men*; 100 μm)-evoked Ca^2+^ entry into transient transfected HEK 293 cells expressing human TRPM8 (*N* = 3/data point, *n*/*N* = 4/12) and full inhibition by M8-B hydrochloride (300 nm). *G*, example Ca^2+^ measurement data from a single 96-well plate showing no effect of 100 nm C31 on allyl isothiocyanate (*AITC*; 30 μm)-evoked Ca^2+^ entry into transiently transfected HEK 293 cells expressing human TRPA1 (*N* = 3/data point, *n*/*N* = 5/15) and full inhibition by A967079 (A96; 5 μm). *Error bars*, S.D.

Based on sequence analysis and functional studies, the closest type of TRP channel to TRPC1/4/5 is mediated by the TRPC3/6/7 cluster of proteins. To investigate C31 against members of these subfamilies, we stably incorporated TRPC3 in HEK 293 cells for tetracycline-inducible expression (Fig. 10*A*). These channels were not activated by EA but could be activated by the diacylglycerol analogue 1-oleoyl-2-acetyl-*sn*-glycerol (OAG), as reported previously (32) (Fig. 10*A*). 100 nm C31 had no effect on TRPC3-mediated Ca^2+^ entry (Fig. 10*A*). Likewise, 100 nm had no effect on OAG-evoked (500 μm) Ca^2+^ entry into transient transfected HEK 293 cells expressing human TRPC6 (Fig. 10*B*). Another subclass of TRP channels is the V class. For this class, we investigated TRPV1 and TRPV4 and again found no effect of 100 nm C31 (Fig. 10, *C* and *D*). We investigated another class of transient receptor potential channels that is activated by H_2_O_2_, TRPM2 channels (33), also expressed using the tetracycline-inducible system in HEK 293 cells (Fig. 10*E*). 100 nm C31 had no effect on TRPM2-mediated Ca^2+^ entry (Fig. 10*E*). Similarly, 100 nm C31 had no effect on another M class subfamily (TRPM8; Fig. 10*F*). To further explore the selectivity of C31, we tested it against TRPA1 and once more found no effect (Fig. 10*G*). The data suggest specificity of C31 for TRPC1/4/5 channels.

### Endogenous channels are sensitive to C31

**Figure 11.**
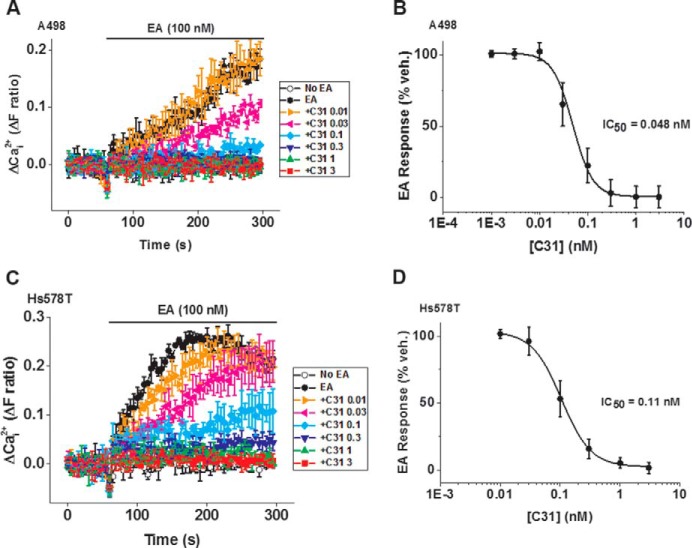
**C31 inhibits endogenous channels in A498 and Hs578T cancer cells.** *A*, example Ca^2+^ measurement data from a 96-well plate showing inhibition of EA (100 nm)-evoked Ca^2+^ entry by 0.01, 0.03, 0.1, 0.3, 1, and 3 nm C31 in A498 cells (*N* = 4/data point). *B*, summary data for experiments of the type shown in *A* as a percentage of the response to EA in the vehicle control for C31 (*n* = 6 independent experiments). The fitted curve is the Hill equation with IC_50_ = 0.048 nm (*n*/*N* = 6/24). *C*, example Ca^2+^ measurement data from a single 96-well plate showing concentration-dependent inhibition of EA (100 nm)-evoked Ca^2+^ entry by 0.01, 0.03, 0.1, 0.3, 1, and 3 nm C31 in Hs578T cells (*N* = 4/data point). *D*, summary data for experiments of the type shown in *C* plotted as a percentage of the response to EA in the vehicle control for C31 (*n* = 5 independent experiments). The fitted curve is the Hill equation with IC_50_ = 0.11 nm (*n*/*N* = 5/20). *Error bars*, S.D.

All of the above data were generated against overexpressed channels. We therefore investigated whether C31 affected endogenous channels, many of which may be heteromeric with TRPC1. Previous studies have suggested that A498 renal cell carcinoma cells contain endogenous EA-activated TRPC1/TRPC4 heteromeric channels (8). To determine the sensitivity of these channels to C31, we measured intracellular Ca^2+^ elevations in response to EA (Fig. 11*A*). C31 (0.3 nm) was sufficient to abolish the Ca^2+^ entry (IC_50_ = 0.048 nm) (Fig. 11, *A* and *B*). Another EA-sensitive cancer cell line (Hs578T cells) was studied, and it was also sensitive to C31 (IC_50_ = 0.11 nm) (Fig. 11, *C* and *D*). The data suggest that endogenous EA-activated channels were inhibited by C31.

### C31 lacks effect on store-operated Ca^2+^ entry

**Figure 12.**
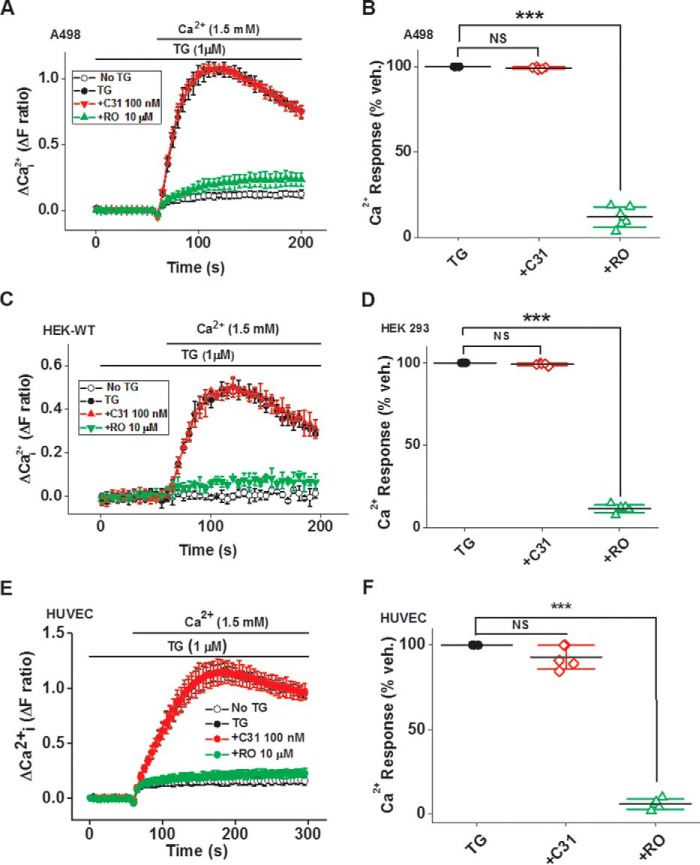
**C31 lacks effect on store-operated Ca^2+^ entry.** *A*, example Ca^2+^ measurement data from a 96-well plate showing effects of 100 nm C31 and 10 μm RO2959 on Ca^2+^ elevation upon adding back extracellular 1.5 mm Ca^2+^ after depletion of intracellular stores with thapsigargin (*TG*; 1 μm) in A498 cells (*n* = 6 for each data point). *B*, summary data for experiments of the same type (*A*) plotted as a percentage of the maximum Ca^2+^ add-back response (*n*/*N* = 6/24). *C*, example Ca^2+^ measurement data from a single 96-well plate showing the effects of 100 nm C31 and 10 μm RO2959 on intracellular Ca^2+^ elevation that occurred upon adding back extracellular 1.5 mm Ca^2+^ after depletion of intracellular Ca^2+^ stores with thapsigargin (*TG*; 1 μm) in HEK 293 cells (*N* = 6/data point). *D*, summary data for *C* (*n*/*N* = 6/24). *E*, as for *C* but in HUVECs. *F*, summary data for experiments of the same type (*E*) plotted as a percentage of the maximum Ca^2+^ add-back response (*n*/*N* = 5/25). *Error bars*, S.D. ***, *p* < 0.001; *NS*, not significant.

Controversial in the Ca^2+^ channel field has been the role of TRPC1/4/5 channels in store-operated Ca^2+^ entry, which is Ca^2+^ entry occurring on adding extracellular Ca^2+^ back to cells that have been store-depleted in Ca^2+^-free medium with the endoplasmic reticulum Ca^2+^-ATPase inhibitor, thapsigargin (9). Therefore, we tested C31 against store-operated Ca^2+^ entry. First, we did this using A498 cells because these cells contained endogenous TRPC1/TRPC4 channels, which were potently inhibited by C31 (Fig. 11, *A* and *B*). There was a large store-operated Ca^2+^ entry into A498 cells, which was strongly inhibited by RO2959 (2,6-difluoro-*N*-5-[4-methyl-1-(5-methyl-thiazol-2-yl)-1,2,5,6-tetrahydro-pyridin-3-yl]-pyrazin-2-yl-benzamide hydrochloride), a known inhibitor of this type of Ca^2+^ entry (Fig. 12, *A* and *B*) (34). Strikingly, 100 nm C31 had no effect on the Ca^2+^ entry (Fig. 12, *A* and *B*). We also investigated the endogenous store-operated Ca^2+^ entry of wild-type HEK 293 cells and human umbilical vein endothelial cells (HUVECs) (Fig. 12, *C–F*). These signals were also inhibited by RO2959 but not C31 (Fig. 12, *C–F*). The data suggest that TRPC1/4/5 channels did not contribute to store-operated Ca^2+^ entry into A498 cells, HEK 293 cells, or HUVECs.

### C31 lacks effect on histamine-evoked Ca^2+^ elevation in endothelial cells

**Figure 13.**
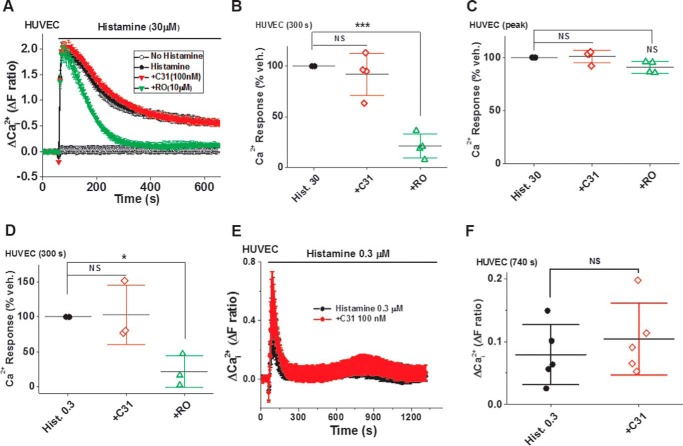
**C31 lacks effect on histamine-evoked Ca^2+^ entry into endothelial cells.** *A*, example Ca^2+^ measurement data from a 96-well plate showing the effects of 100 nm C31 and 10 μm RO2959 on Ca^2+^ elevation in response to 30 μm histamine in the continuous presence of extracellular 1.5 mm Ca^2+^ in HUVECs (*N* = 6/data point). Summary data for experiments of the same type (*A*) measured 300 s (*B*) after the start of histamine application and on the peak (*C*) response (*n*/*N* = 4/16). *D*, as for *A* but using 0.3 μm histamine and showing only summary data (*n*/*N* = 3/12). *E*, example Ca^2+^ data from a 96-well plate showing the effect of 100 nm C31 on Ca^2+^ elevation during a 22.5-min response to 0.3 μm histamine in the presence of extracellular 1.5 mm Ca^2+^ in HUVECs (*n* = 6 for each data point). *F*, summary data for experiments of the same type (*E*) measured 740 s after the start of histamine application and plotted as a percentage of the maximum Ca^2+^ add-back response (*n*/*N* = 6/36). *Error bars*, S.D. *, *p* < 0.05; ***, *p* < 0.001; *NS*, not significant.

**Figure 14.**
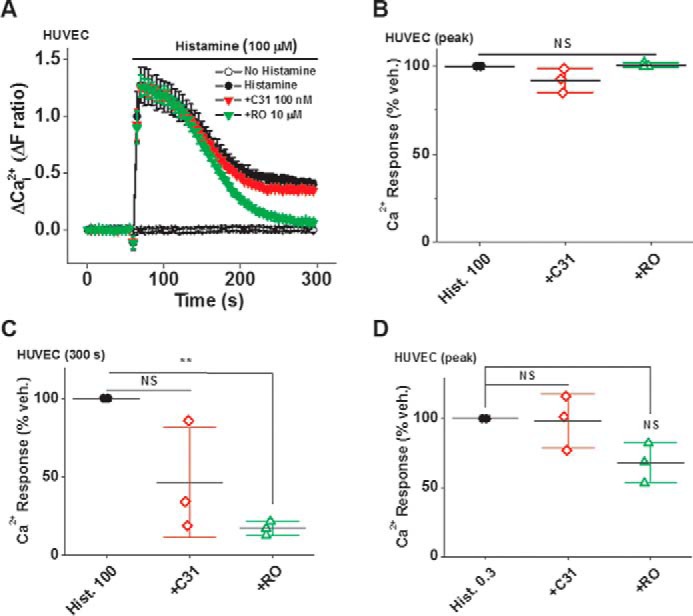
**Additional data for the effects of C31 and RO2959 on histamine-evoked Ca^2+^ entry into endothelial cells.** *A*, example Ca^2+^ measurement data from a 96-well plate showing effects of 100 nm C31 and 10 μm RO2959 on Ca^2+^ elevation in response to 100 μm histamine in the continuous presence of extracellular 1.5 mm Ca^2+^ (*N* = 6/data point). Summary data for experiments of the same type (*A*) measured at the peak response (*B*) and 300 s (*C*) after the start of histamine application (*n*/*N* = 3/18). *D*, summary Ca^2+^ measurement data for the effects of 100 nm C31 and 10 μm RO2959 on the peak Ca^2+^ elevation in response to 0.3 μm histamine in the continuous presence of extracellular 1.5 mm Ca^2+^ in HUVECs (*n*/*N* = 6/36). *Error bars*, S.D. **, *p* < 0.01; *NS*, not significant.

Store-operated Ca^2+^ entry evoked by the thapsigargin Ca^2+^ addback protocol may not be the same as Ca^2+^ entry caused by a physiological agonist. We therefore investigated the effect of histamine on HUVECs in the continuous presence of extracellular Ca^2+^ (Figs. 13 and 14). Histamine (0.3–100 μm) caused a fast rise in intracellular Ca^2+^, which then declined to a plateau (Figs. 13 and 14). The initial rise is thought to reflect Ca^2+^ release from intracellular stores, which may cause at least partial depletion of intracellular Ca^2+^ stores. C31 had no effect, whereas RO2959 strongly suppressed the plateau but not the initial rise (Figs. 13 and 14). When the response to 0.3 μm histamine was studied over a longer period, a second phase of Ca^2+^ elevation was observed (Fig. 13, *E* and *F*). This signal was also not inhibited by C31 and even showed a tendency to be potentiated (Fig. 13, *E* and *F*). The data suggest that TRPC1/4/5 channels did not contribute positively to histamine-evoked Ca^2+^ release or entry into HUVECs.

## Discussion

The data suggest superb effectiveness, potency, and specificity of C31 as an inhibitor of channels formed by TRPC1/4/5 proteins. Importantly, C31 inhibited heteromeric channels involving TRPC1 and had graded potency and differential characteristics against channels formed by different combinations of TRPC1/4/5 (Table 1).

**Table 1:** **Summary of IC_50_ values in pm and ranked from lowest to highest** The channel type studied is indicated on the left. TRPC4, TRPC5, TRPC4-TRPC1, and TRPC5-TRPC1, human channel constructs expressed in HEK 293 cells. Fura-2 (Ca^2+^), FlexStation 96-well recordings. Patch, whole-cell voltage-clamp recordings with current measured at the indicated voltage (in some cases, the current was measured at −90 or +90 mV instead of −100 or +100 mV because the current amplitude saturated the amplifier in a small number of these experiments).

Channel	Assay	Agonist	C31 IC_50_
			*pm*
TRPC4-TRPC1	Patch − 100 mV	200 nm S1P	9
TRPC4-TRPC1	Fura-2 (Ca^2+^)	200 nm S1P	11
TRPC4	Patch − 100 mV	200 nm S1P	12
TRPC4	Patch + 100 mV	200 nm S1P	30
TRPC4-TRPC1	Fura-2 (Ca^2+^)	10 nm EA	33
TRPC4-TRPC1	Patch + 90 mV	10 nm EA	42
A498 endogenous	Fura-2 (Ca^2+^)	100 nm EA	48
TRPC4-TRPC1	Patch + 100 mV	200 nm S1P	59
TRPC4-TRPC1	Patch − 90 mV	30 nm EA	61
TRPC4	Patch − 90 mV	10 nm EA	63
TRPC4-TRPC1	Patch − 90 mV	10 nm EA	68
TRPC4-TRPC1	Patch + 90 mV	30 nm EA	78
Hs578T endogenous	Fura-2 (Ca^2+^)	100 nm EA	110
TRPC4	Patch + 90 mV	10 nm EA	169
TRPC5-TRPC1	Fura-2 (Ca^2+^)	10 nm EA	199
TRPC4	Fura-2 (Ca^2+^)	10 nm EA	349
TRPC4-TRPC1	Patch + 90 mV	100 nm EA	441
TRPC4-TRPC1	Patch − 90 mV	100 nm EA	481
TRPC4	Patch − 90 mV	100 nm EA	593
TRPC4	Patch + 90 mV	100 nm EA	916
TRPC5	Fura-2 (Ca^2+^)	10 nm EA	1300

It has been challenging to find selective and potent pharmacological agents for the modulation of TRPC1/4/5 channels (10). Small-molecule inhibitors, such as SKF-93635 and 2-APB, are nonspecific. ML204 and the anti-histamine clemizole hydrochloride were more recently identified and are more specific, but they only inhibit the channels at micromolar concentrations (13, 35). We show here that the IC_50_ for ML204 against the TRPC4-TRPC1 heteromeric channel is 58 μm. There are also other less potent compounds compared with ML204, namely M084-derived compounds, which inhibit TRPC4/TRPC5 channels (36). Galangin is a natural product recently identified as an inhibitor of TRPC4/5 channels, but micromolar concentrations were needed, the compound was not specific, and the effect against TRPC1-containing channels was weak (37). Small-molecule activators of TRPC1/4/5 channels have been identified and include riluzole and rosiglitazone, but they are nonspecific, and high concentrations are needed for an effect (38, 39). EA was the first identified remarkable small-molecule modulator of TRPC1/4/5 channels (8, 11). It is an agonist with nanomolar potency, good specificity, and strong efficacy. C31 is an inhibitor that is also remarkable, perhaps more so than EA. It is even more potent than EA and also appears to be highly specific. Importantly, it had better effect on TRPC1-containing heteromers, and there was subtype specificity over 2 orders of magnitude concentration range, all in the picomolar to low-nanomolar range. Because of the remarkable and unprecedented potency of C31 as a TRPC1/4/5 inhibitor, we validated its effect using three different batches of C31, made using three independent synthetic routes (see supplemental Information). All three batches were analytically pure according to NMR and LC-MS analysis (supplemental Information and Figs. S5–S7) and displayed the same activity against TRPC4-TRPC1 concatemers (supplemental Information and Fig. S8), validating C31 as the active compound.

The detailed mechanism of action of C31 remains to be elucidated. We do not have evidence that C31 directly binds the channels, but our data suggest a direct effect via the extracellular surface of the channels or extracellular leaflet of the bilayer. The effect of C31 was not as rapid as might be expected for an agent that plugs the ion pore. Voltage dependence in the action of C31 was relatively mild, also not supporting the idea of blockade deep in the ion pore and electric field. Moreover, an agonistic effect of C31 was observed in the presence of Gd^3+^, suggesting that C31 might be a modulator. Intriguingly, C31 had no agonist effect in the absence of Gd^3+^, indicating that Gd^3+^ exposed a different mode of action of C31 or an effect that depended on the prior gating state. The effect of C31 was not dependent on EA, yet it was modulated by EA. There is no firm evidence that EA directly binds the channels, but it is effective in outside-out patches, so it apparently acts directly (8). Because of the differences in the chemistry of EA and C31, we suggest that it is likely that elevated EA concentration reduces the inhibitory potency of C31 via allosteric modulation (*i.e.* that EA and C31 bind different sites).

C31 was more potent against TRPC4-mediated ionic current than TRPC4-mediated Ca^2+^ entry: IC_50_ = 0.063–0.169 nm *versus* 0.349 nm (activation by 10 nm EA) (Table 1). This difference was not evident for TRPC4-TRPC1 channels: IC_50_ = 0.068–0.042 nm *versus* 0.033 nm (activation by 10 nm EA) (Table 1). We speculate that the differential effect was caused by technical differences in the electrophysiology and Ca^2+^ measurement experiments combined with differences in the effects of C31 on TRPC4 and TRPC4-TRPC1 channels. Voltage dependence was most obvious in the potency of C31 against TRPC4, and the membrane potential was not voltage-clamped in the Ca^2+^ measurement studies. However, this explanation is not quantitatively sufficient to explain the difference, so it might be that the potency of C31 against TRPC4 depends on additional unknown factors.

C31 was more potent than we expected against endogenous EA-evoked Ca^2+^ entry into A498 cells. We used 100 nm EA in these experiments to ensure a robust Ca^2+^ response. In patch-clamp recordings, this concentration of EA reduced the IC_50_ for C31 against TRPC4-TRPC1 channels to about 0.45 nm, whereas in A498 cells, the IC_50_ for C31 against Ca^2+^ entry was about 10 times lower (Table 1). There are various possible explanations, including that the TRPC4-TRPC1 concatemer did not perfectly mimic the endogenous channels of A498 cells and that channel density affected potency.

The first publications on TRPC1/4/5 suggested roles in Ca^2+^ entry triggered by Ca^2+^ store depletion (*i.e.* that these proteins were subunits of store-operated Ca^2+^ channels, capacitative Ca^2+^ entry, or Ca^2+^ release-activated Ca^2+^ (CRAC) channels) (40–42). Ever since, debate has continued about whether they do indeed serve this role. A challenge came with the discovery of the Orai1 protein, a previously unrecognized type of Ca^2+^ channel subunit that could reliably reproduce the properties of CRAC channels if co-expressed with STIM1 protein (9, 43). Depletion of Orai1 by short interfering RNA suppressed store-operated Ca^2+^ entry in the hands of many investigators. Whereas depletion of TRPC1/4/5 (especially TRPC1) has also suppressed store-operated Ca^2+^ entry, some investigators have not been able to observe this effect in their cells and experimental conditions. Prominent publications have suggested interactions between STIM1 and the TRPC1/4/5 proteins (44, 45). There seems now to be broad agreement that TRPC1/4/5 proteins do not contribute to CRAC channels, but the topics of other less Ca^2+^-selective channels activated by store depletion and the phenomenon of Ca^2+^ entry in store-depleted cells are broader, and there is less clarity about the roles of TRPC1/4/5. We suggest that C31 could be a useful tool for investigating these roles in many cell and tissue types and experimental conditions, all of which might have a bearing on whether TRPC1/4/5 proteins contribute. We investigated C31 against store-operated Ca^2+^ entry in three cell types as examples. Strikingly, in all cases, C31 had no effect on the store-operated Ca^2+^ entry at a concentration orders of magnitude greater than that needed to inhibit TRPC1/4/5 channels. We suggest that these data exclude the contribution of TRPC1/4/5 channels to store-operated Ca^2+^ entry in these cells under these conditions. There should be caution, however, because store depletion causes clustering of membrane proteins and potentially new assemblies of proteins that are not otherwise present; if these new assemblies involve TRPC1/4/5 proteins forming Ca^2+^-permeation pathways with other proteins, these assemblies might then not be sensitive to C31.

In conclusion, the study suggests C31 as a promising tool for critical evaluation of TRPC1/4/5 channels in biological mechanisms and that store-operated Ca^2+^ entry can occur in a cell without any contribution from TRPC1/4/5 channels although functional channels exist in the same cell. Because of the special effects of C31, we suggest the name Pico145.

## Materials and methods

### Cell culture

HEK 293 cells stably expressing tetracycline-regulated human TRPC3, TRPC4, TRPC5, or TRPM2 have been described (8, 33, 37, 46). All cells were grown at 37 °C in a 5% CO_2_ incubator and culture media supplemented with fetal bovine serum (FBS; 10%), penicillin (50 units/ml), and streptomycin (0.5 mg/ml) (Sigma-Aldrich). The modified HEK 293 cells were maintained in Dulbecco's modified Eagle's medium/F-12 GlutaMAX supplemented with selection antibiotics blasticidin (5 μg/ml) and Zeocin (400 μg/ml) (Invitrogen). To induce expression of channels in these modified HEK 293 cells, 1 μg/ml tetracycline was added to the media 24 h before experiments. A498 cells and Hs578T cells were maintained in minimum Eagle's medium with Earle's balanced salt solution, l-glutamine, 2.2 g/liter NaHCO_3_ (PAN-Biotech, Aidenbach, Germany) and in RPMI 1640 GlutaMAX (Invitrogen), respectively. HUVECs were cultured in EGM-2 growth medium supplemented with an EGM-2 bullet kit (Lonza). TRPV4 was studied in Chinese hamster ovary (CHO) K1 cells stably expressing human TRPV4 and maintained in Ham's F-12 (Gibco) in the presence of 1 mg/ml G418 (Sigma).

### Transfected HEK 293 cells

Transfections of HEK 293 cells with human TRPC6, TRPA1, TRPV1, and TRPM8 (vector; pcDNA3.1) were performed using FuGENE HD transfection reagent (Roche Applied Science). The HEK 293 cells were split and plated, 5 × 105 cells/well, in a 6-well culture dish 24 h before transfection in 2 ml of DMEM/F-12 GlutaMAX medium. Next the cells were transfected using a ratio of 3:1 ml of FuGENE HD/μg of total cDNA (stock (1 μg/μl). The transfection reaction was done in a 1.5-ml microcentrifuge tube for each well, and the following materials were added to the reaction: 100 μl of Opti-MEM (Gibco; 37 °C), 1 μg of human cDNA, and finally 3 μl of the FuGENE HD transfection reagent. Transfection reactions were mixed vigorously by vortexing and incubated at room temperature for 15 min. Finally, the reactions were added to each well, and the 6-well plate was incubated at 37 °C in a 5% CO_2_. The next day, the transfected cultured cells were removed to 96-well clear-bottom poly-d-lysine-coated black plates (Corning Life Sciences, Lowell, MA) and incubated, additionally, overnight for the Ca^2+^ measurement.

### Generation of TRPC4-TRPC1 and TRPC5-TRPC1 concatemers

Human TRPC4 and TRPC1 were cloned upstream and downstream, respectively, of a four-amino acid linker (ASAS), flanked by AgeI and SacII restriction endonuclease sites, that had previously been introduced into pcDNA^TM^ 4/TO (8). TRPC4 β, including an N-terminal Kozak sequence, was inserted between BamHI and AgeI restriction sites using hTRPC4 β/pcDNA^TM^ 4/TO (8), a PCR template (forward primer, 5′-AGTCGGATCCGCCACCATGGCTCAGTTCTATTACAAAAG-3′; reverse primer, 5′-AGTTACCGGTCAATCTTGTGGTCACGTAATCTTC-3′). TRPC1 was inserted between SacII and XbaI restriction sites using hTRPC1/pIRES as a PCR template (forward primer, 5′-ACTCCGCGGCATGATGGCGGCCCTG-3′; reverse primer, 5′-AGTCTCTAGATTAATTTCTTGGATAAAACATAGCATATTTAG-3′). HEK 293 cells stably expressing the TRPC5-TRPC1 and TRPC4-TRPC1 constructs were then generated for tetracycline-regulated expression as for TRPC5 HEK 293 Tet cells (46).

### Intracellular Ca^2+^ measurement

Cells were seeded at 90% confluence into 96-well clear-bottomed poly-d-lysine-coated black plates (Corning Life Sciences) for HEK 293 cells and clear-bottomed Nunc plates (Thermo Scientific) for A498 cells, Hs578T cells, and HUVECs 24 h before experimentation. Fura-2 Ca^2+^ indicator dye was used to monitor changes in intracellular ionized Ca^2+^ concentration. To perform the experiment, the cells were incubated for 1 h with fura-2-AM (2 μm) in standard bath solution (SBS) at 37 °C in the presence of 0.01% pluronic acid. SBS contained 135 mm NaCl, 5 mm KCl, 1.2 mm MgCl_2_, 1.5 mm CaCl_2_, 8 mm glucose, and 10 mm Hepes (pH titrated to 7.4 using NaOH). Subsequently, the cells were washed twice with SBS before adding C31 or ML204 for 30 min before making Ca^2+^ measurements. The fura-2 fluorescence was recorded using a 96-well fluorescence plate reader and the excitation wavelengths of 340 and 380 nm (FlexStation II^384^, Molecular Devices, Sunnyvale, CA). For TRPV4 recordings, fluo-4/AM was used in place of fura-2/AM, and 2.5 mm probenecid was included to inhibit leak of fluo-4. Fluo-4 was excited at 485 nm, and emitted light was collected at 525 nm. Ca^2+^ was indicated by the ratio of the fluorescence (F) emission intensities for the two excitation wavelengths. Measurements were made at room temperature (21 ± 3 °C).

### Patch-clamp recording

Conventional whole-cell configuration and outside-out patch-clamp recordings were performed under voltage clamp at room temperature using 2–4-megaohm patch pipettes fabricated from borosilicate glass capillaries with an outside diameter of 1 mm and an inside diameter of 0.58 mm (Harvard Apparatus). The currents were recorded using an Axopatch 200B amplifier, digitized by a Digidata 1440, and recorded to computer using pCLAMP10 (Molecular Devices). The data were filtered at 1 kHz and analyzed off-line using Clampfit software (version 10.2, Molecular Devices) and Origin software version 9.1 (OriginLab, Northampton, MA). The bath solution was SBS, and the pipette solution (intracellular solution) contained 145 mm CsCl, 2 mm MgCl_2_, 10 mm Hepes, 1 mm EGTA (free acid), 5 mm ATP (sodium salt), and 0.1 mm Na·GTP (sodium salt), titrated to pH 7.2 with CsOH. Cells were plated 24 h previously on glass coverslips at a low density of 20–30% and induced with tetracycline (1 μg/ml).

### C31 synthesis

The compound was synthesized as described previously (28).

### Chemicals and reagents

EA was prepared as described previously (8). C31 was stored at −20 °C as a 10 mm stock. All commercial chemicals used in this work were purchased from Sigma-Aldrich, except where stated. RO2959 was from Aobious Inc. Fluo-4/AM was from Life Technologies. For stocks, all chemicals were dissolved in 100% DMSO except S1P, which was dissolved in methanol at 5 mm and Gd^3+^ (GdCl_3_) in deionized water at 100 mm.

### Data analysis

Origin software was used to analyze and present the results. Concentration-response curves were fitted using the Hill equation. The data points are shown using scatter plots, and S.D. values are represented as *error bars*. The number of independent experiments is indicated by *n*, and the number of replicates within an independent experiment is indicated as *N* (*e.g.* wells in a 96-well plate). Tests of significance were performed using a paired two-sample *t* test, and statistical significance is indicated by an *asterisk* (*, *p* < 0.05; **, *p* < 0.01; ***, *p* < 0.001).

## Author contributions

H. N. R. performed the calcium measurement studies that initially identified the potency of the inhibitor, contributed intellectually, analyzed data, generated figures, and wrote parts of the manuscript. M. J. L., K. E. M., and N. M. B. generated the concatemers and stable cell lines. K. Muraki contributed intellectually, generated figures, analyzed data, and performed the patch-clamp experiments supported by H. N. R., S. Y. C., and Y. T., and M. H., N. H., R. T., R. F., R. S. B., and M. C. performed or advised on chemical synthesis and usage. H. J. G. and K. Miteva performed histamine and HUVEC cell experiments. H. L. A., M. A. B., L. M., H. W., and P. N. provided intellectual input or technical advice. All authors commented on the manuscript. D. J. B. initiated the project, generated research funds and ideas, led and coordinated the project, interpreted data, and wrote most of the paper.

## Supplementary Material

Supplemental Data

## Footnotes

4 The abbreviations used are:

TRPC: transient receptor potential canonical
OAG: 1-oleoyl-2-acetyl-*sn*-glycerol
EA: (−)-englerin A
C31: compound 31
HEK: human embryonic kidney
IV: current-voltage relationship
S1P: sphingosine 1-phosphate
HUVEC: human umbilical vein endothelial cell
CRAC: Ca^2+^ release-activated Ca^2+^
SBS: standard bath solution.
