# Supplementary material for: Picomolar, selective, and subtype-specific small-molecule inhibition of TRPC1/4/5 channels
Source: J Biol Chem. 2017 Mar 21;292(20):8158–73. doi: 10.1074/jbc.M116.773556 (PMC5437225; doi:10.1074/jbc.M116.773556)
Supplement: Supplemental Data [file supp_292_20_8158__index.html]

Picomolar, selective and subtype specific small-molecule inhibition of TRPC1/4/5 channels — Picomolar, selective, and subtype-specific small-molecule inhibition of TRPC1/4/5 channels — TRPC1/4/5 inhibitor — Supplemental Data 

# Picomolar, selective, and subtype-specific small-molecule inhibition of TRPC1/4/5 channels

## Supplemental Data

- Supplementary Information (.pdf, 1.5 MB) - Supplementary Information 3 Schemes (S1-S3) 8 Figures (Figures S1-S8)
