# Supplementary material for: Picomolar, selective, and subtype-specific small-molecule inhibition of TRPC1/4/5 channels
Source: J Biol Chem. 2017 Mar 21;292(20):8158–73. doi: 10.1074/jbc.M116.773556 (PMC5437225; doi:10.1074/jbc.M116.773556)
Supplement: Supplemental Data [file 10.1074_M116.773556_jbc.M116.773556-1.pdf]

SUPPLEMENTARY INFORMATION

3 Schemes (S1-S3)
8 Figures (Figures S1-S8)

Picomolar, selective and subtype specific small-molecule inhibition of TRPC1/4/5 channels

Hussein N Rubaiy[1], Melanie J Ludlow[1], Matthias Henrot[2], Hannah J Gaunt[1], Katarina Miteva[1], Sin Yin Cheung[1], Yasuyuki Tanahashi[1,3], Nurasyikin Hamzah[4], Katie E Musialowski[1], Nicola M Blythe[1], Hollie L Appleby[1], Marc A Bailey[1], Lynn McKeown[1], Roger Taylor[4], Richard Foster[4], Herbert Waldmann[5], Peter Nussbaumer[6], Mathias Christmann[2], Robin S Bon[1], Katsuhiko Muraki[7*], David J Beech[1*]

Schools of [1]Medicine and Chemistry[4], University of Leeds, Leeds, LS2 9JT, UK. [2]Institute of Chemistry and Biochemistry, Freie Universität Berlin, Takustraße 3, 14195 Berlin, Germany. [3]Faculty of Life Sciences, Kyoto Sangyo University, Kyoto 603-8555, Japan. [5]Max-Planck-Institut für Molekulare Physiologie, Otto-Hahn-Straße 11, 44227 Dortmund, Germany. [6]Lead Discovery Center GmbH, Otto-Hahn-Str. 15, D-44227 Dortmund, Germany. [7]School of Pharmacy, Aichi-Gakuin University, 1-100 Kusumoto, Chikusa, Nagoya 464-8650, Japan.

Running title: *TRPC1/4/5 inhibitor*

*Authors for correspondence: Professor David J Beech, Leeds Institute of Cardiovascular and Metabolic Medicine, School of Medicine, LIGHT Building, Clarendon Way, University of Leeds, Leeds LS2 9JT, UK. Telephone +44 (0) 113 343 4323. Email d.j.beech@leeds.ac.uk. Professor Katsuhiko Muraki, School of Pharmacy, Aichi-Gakuin University, 1-100 Kusumoto, Chikusa, Nagoya 464-8650, Japan. Email kmuraki@dpc.aichi-gakuin.ac.jp

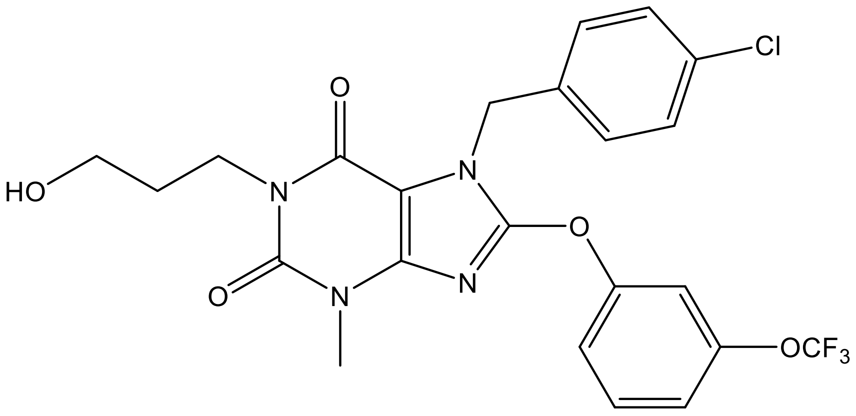

Figure S1. Chemical structure of Compound 31 (C31).

Details of chemical synthesis and validation of active ingredient

Because of the remarkable and unprecedented potency of compound **C31** as a TRPC1/4/5 inhibitor, we validated the identity of the active compound as follows: compound **C31** was prepared according to three independent synthetic routes, by three different investigators, at two different institutes (FU Berlin and University of Leeds), and the identity, purity and activity of the three batches were compared. Experimental details, full characterisation of **C31**, spectra and assay data are shown below.

Scheme S1. Synthetic route 1 (Berlin)

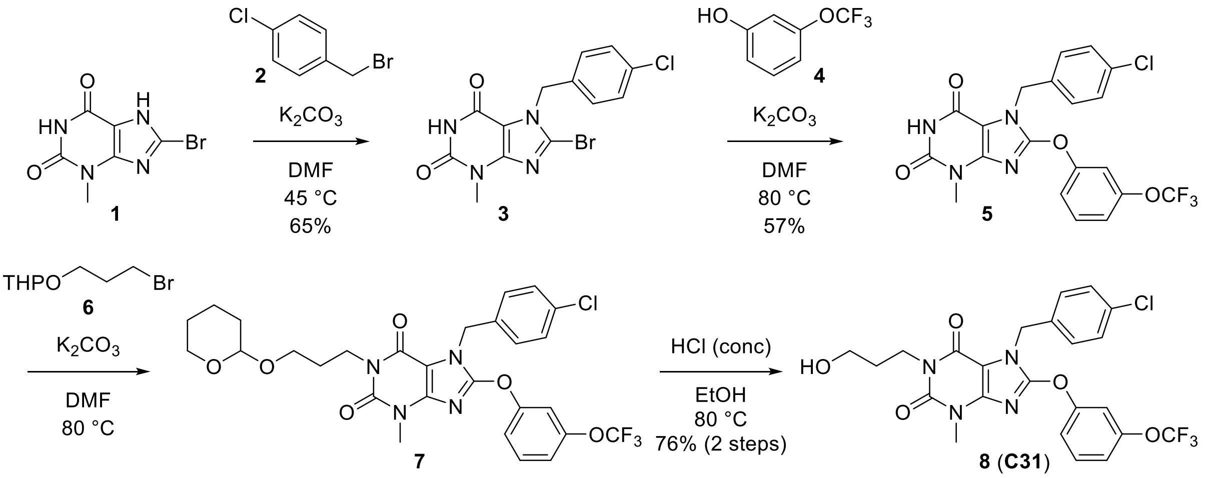

Compound 3: To a solution of 8-bromo-3-methyl-1*H*-purine-2,6(3*H*,7*H*)-dione **1** (1.65 g, 6.73 mmol, 1 equiv.) in DMF (30 mL) was added 1-(bromomethyl)-4-chorobenzene **2** (1.52 g, 7.40 mmol, 1.1 equiv.) followed by K_2CO_3 (1.40 g, 10.10 mmol, 1.5 equiv.). The resulting mixture was heated at 45 °C for 2 h and additional K_2CO_3 (1.40 g, 10.10 mmol, 1.5 equiv.) was added. After 1h, the mixture was diluted with EtOAc (70 mL) and washed with brine (50 mL). The layers were separated and the organic slurry was filtered and the cake was washed with ice-cold ethanol leading to **3** (1.61 g, 65 %) as a white solid. Analytical data were in agreement with data reported in patent WO 2014143799.

Compound 5: To a solution of compound **3** (500 mg, 1.35 mmol, 1 equiv.) in DMF (6 mL) in a 10 mL conical flask was added 3-(trifluoromethoxy)phenol **4** (262 µL, 361 mg, 2.03 mmol, 1.5 equiv.) followed by K_2CO_3 (802 mg, 5.81 mmol, 4.3 equiv.). The flask was sealed and the resulting mixture was heated at 80 °C overnight (the flask was entirely immersed in the heating bath). The mixture was then partitioned between water (50 mL) and EtOAc (80 mL). The aqueous phase was extracted with EtOAc (2 x 80 mL) and the combined organic layers were dried (Na_2SO_4) and concentrated in vacuo. The resulting white solid was washed with ethanol

in a sinter leading to **5** (360 mg, 57 %) as a white solid. Analytical data were in agreement with data reported in patent WO 2014143799.

Compound 7: To a solution of compound **5** (300 mg, 0.643 mmol, 1 equiv.) in DMF (3.75 mL) in a 10 mL conical flask was added 2-(3-bromopropoxy)tetrahydro-*2H*-pyran **6** (215 mg, 0.964 mmol, 1.5 equiv.) followed by K_2CO_3 (267 mg, 1.929 mmol, 3 equiv.). The flask was sealed and the resulting mixture was heated at 80 °C overnight (the flask was entirely immersed in the heating bath). The mixture was then partitioned between water (20 mL) and EtOAc (20 mL). The aqueous phase was extracted with EtOAc (2x20 mL) and the combined organic layers were dried (Na_2SO_4) and concentrated in vacuo leading to compound **7** as a white solid, which was immediately used in the next reaction.

Compound 8 (C31): All of compound **7** was dissolved in EtOH (5.0 mL) in a 100 mL round bottom flask and concentrated HCl (1 mL) was added. The mixture was stirred overnight at 80 °C in a sealed flask. The reaction was quenched with saturated aqueous $NaHCO_3$ (20 mL) and the aqueous phase was extracted with EtOAc (3x20 mL). The combined organic layers were dried (Na_2SO_4) and concentrated in vacuo. The residue was purified using flash chromatography (pentane/EtOAc, 1:1) to give **8** (**C31**) (256 mg, 76 % over 2 steps). Analytical data were in agreement with data reported in patent WO 2014143799 (see below).

Scheme S2. Synthetic route 2 (Leeds)

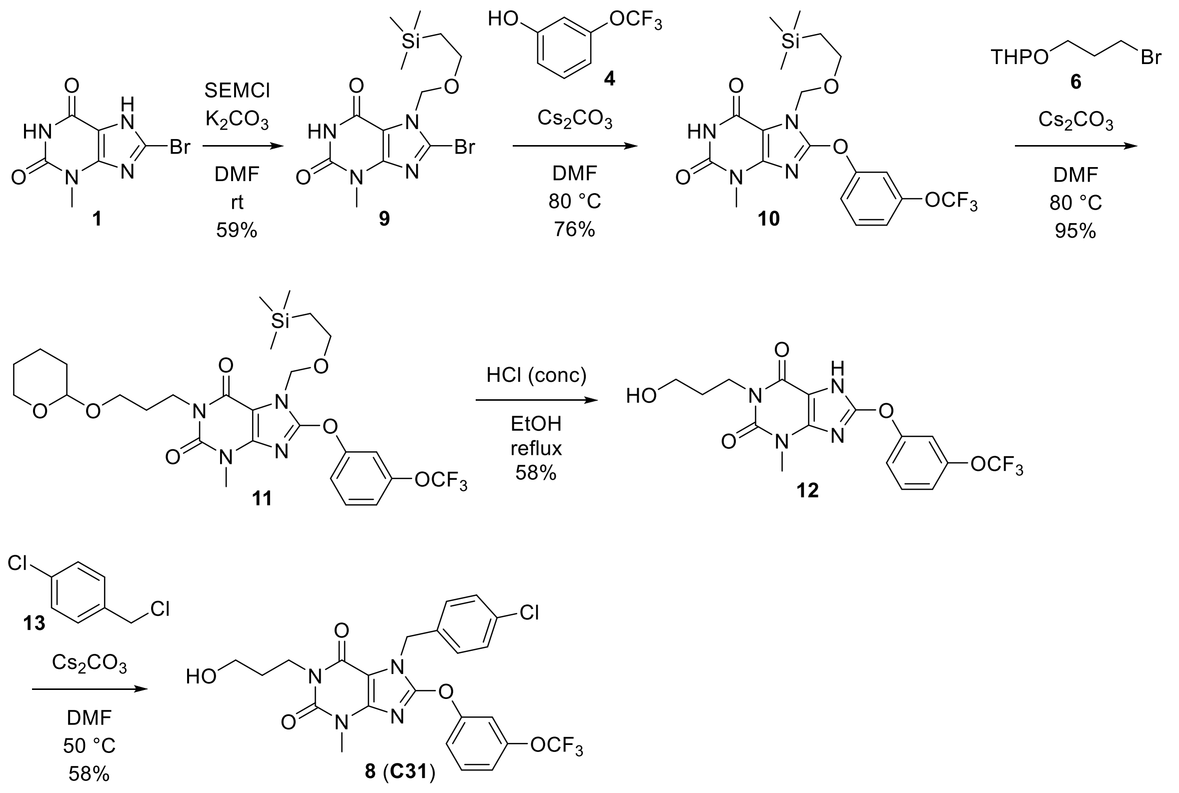

Compound 9: To a solution containing 8-bromo-3-methyl-1*H*-purine-2,6(3*H*,7*H*)-dione **1** (1.0 g, 4.12 mmol) and K_2CO_3 (1.1 g, 8.23 mmol) in DMF (10 mL), 2-(trimethylsilyl)ethoxymethyl chloride (0.73 mL, 4.12 mmol) was added dropwise at 0 °C. After 3 hours of stirring at rt, aqueous LiCl solution (10%, 1 mL) was added and the mixture was partitioned between EtOAc (25 mL) and water (25 mL). The layers were separated and the aqueous layer was extracted with EtOAc (25 mL). The combined organic layers were washed with brine (25 mL), dried ($MgSO_4$), filtered and evaporated to dryness. The white crude product was triturated with cold EtOH and the white solid **9** was dried overnight and was used without further purification (920 mg, 59%). Analytical data were in agreement with data reported in patent WO 2014143799.

Compound 10: To a solution of compound **9** (800 mg, 2.14 mmol) in DMF (10 mL) was added Cs_2CO_3 (2.1 g, 6.42 mmol) and 3-(trifluoromethoxy)phenol **4** (0.27 mL, 2.1 mmol). The mixture was heated at 80 °C overnight. The mixture was cooled and aqueous LiCl solution (10%, 1 mL) was added and the mixture was partitioned between EtOAc (10) and water (10 mL). The organic layer was washed with brine (10 mL), dried ($MgSO_4$), filtered and evaporated to dryness. The crude product was triturated with EtOH to afford white solid product **10** which was used without further purification (760 mg, 76%). Analytical data were in agreement with data reported in patent WO 2014143799.

Compound 11: To a solution of compound **10** (660 mg, 1.4 mmol) in DMF (5 mL) was added 2-(3-bromopropoxy)tetrahydro-2*H*-pyran **6** (370 mg, 1.9 mmol) and Cs_2CO_3 (1.37 g, 4.2 mmol).The reaction was heated at 50 °C for 3 hours. The mixture was cooled, partitioned between EtOAc (10 mL) and water (10 mL). The organic layer was dried ($MgSO_4$), filtered and concentrated to give a crude product which was purified by flash column chromatography by eluting with 0-100% EtOAc in Pet. Ether. The product fractions were combined and the solvent was evaporated to afford **11** as a white solid (820 mg, 95%). Analytical data were in agreement with data reported in patent WO 2014143799.

Compound 12: To a solution of compound **11** (800 mg, 1.3 mmol) in ethanol (10 mL) was added 37% concentrated HCl (1.8 mL) and the reaction was refluxed for 24 hours until all starting material was converted to product. Upon completion, the reaction mixture was concentrated in vacuo to give a crude product, which was purified using flash column chromatography by eluting with 0-100% EtOAc in Pet.Ether. The product fractions were combined and the solvent was evaporated to afford **12** as a white solid (300 mg, 58%). Analytical data were in agreement with data reported in patent WO 2014143799.

Compound 8 (C31): To a solution of compound **12** (300 mg, 0.75 mmol) in DMF (5 mL) was added 1-(chloromethyl)-4-chorobenzene **13** (180 mg, 1.13 mmol) followed by Cs_2CO_3 (500 mg, 1.5 mmol). The resulting mixture was stirred at 50 °C for 3 hours. Aqueous LiCl solution

(10%, 1 mL) was added and the mixture was partitioned between EtOAC (10 mL) and water (10 mL). The organic layer was washed with brine (10 mL), dried ($MgSO_4$), filtered and evaporated to dryness. The crude product was purified using flash chromatography by eluting with 0-100% EtOAc in Pet. Ether. The product fractions were combined and the solvent was evaporated to afford the **8** (**C31**) as a white solid (270 mg, 69%). Analytical data were in agreement with data reported in patent WO 2014143799 (see below).

Scheme S3. Synthetic route 3 (Leeds)

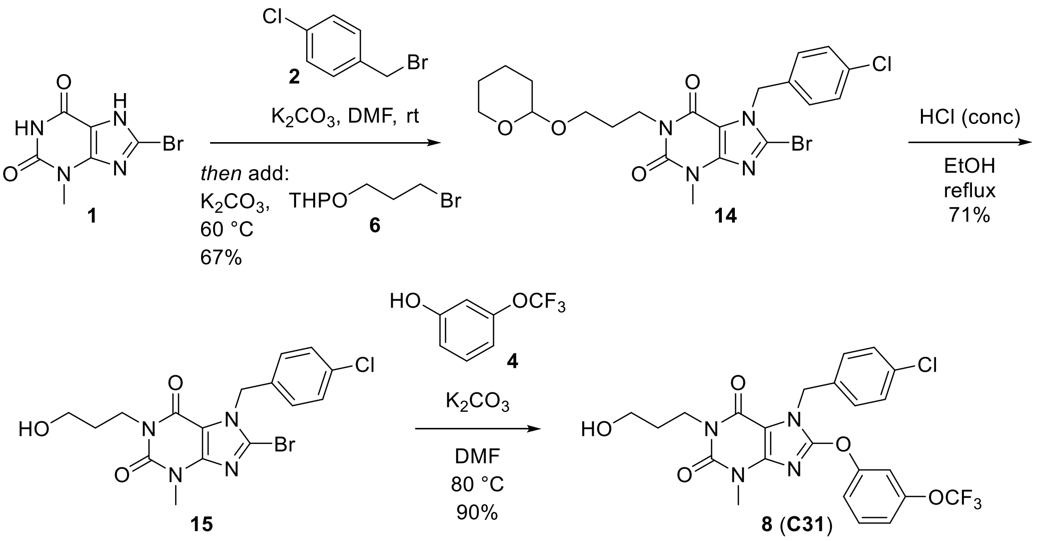

Compound 14: To a suspension of 8-bromo-3-methyl-1*H*-purine-2,6(3*H*,7*H*)-dione **1** (3.0 g, 12.2 mmol) and K_2CO_3 (1.69 g, 12.2 mmol) in DMF (30 mL), 1-(bromomethyl)-4-chorobenzene **2** (2.50 g, 12.2 mmol) was added. The mixture was stirred at rt for 1.5 hours. Then, another equivalent of K_2CO_3 (1.69 g, 12.2 mmol) and 2-(3-bromopropoxy)tetrahydro-2*H*-pyran **6** (2.14 mL, 12.81 mmol) were added and the reaction mixture was stirred at 60 °C overnight. The reaction mixture was cooled to rt, poured into water (200 mL), and extracted with EtOAc (4 x 200 mL). The combined organic layers were washed with aqueous LiCl solution (1N, 2 x 100 mL), brine (100 mL), dried with Na_2SO_4 and concentrated in vacuo. The crude product was dissolved in boiling MeCN (80 mL), and water was added (40 mL) while hot. The solution was cooled slowly to rt (overnight). Crystals were collected and washed with water (30 mL), cold EtOH (30 mL) and dried in vacuo to give **14** as a fluffy white powder (4.18 g, 67%). Analytical data were in agreement with data reported in patent WO 2014143799.

Compound 15: Compound **14** (4.15 g, 8.08 mmol) was suspended in EtOH (70 mL) and concentrated aqueous HCl (10 mL) was added. The mixture was heated at reflux for 1.5 h (all solid dissolves). After cooling, the reaction mixture was concentrated to dryness in vacuo. The

crude product was purified by flash column chromatography using 0-40% EtOAc in DCM, affording **15** as a white solid (2.45 g, 71%). Analytical data were in agreement with data reported in patent WO 2014143799.

Compound 8 (C31): To a suspension of compound **15** (1.5 g, 3.5 mmol) and K_2CO_3 (967 mg, 7 mmol) in DMF (15 mL) was added 3-(trifluoromethoxy)phenol **4** (0.475 mL, 3.7 mmol), and the reaction mixture was stirred at 80 °C for 4 h. After cooling to rt, the reaction mixture was diluted with water (200 mL) and extracted with EtOAc (200 mL, then 3 x 100 mL). The combined organic layers were washed with aqueous LiCl solution (1N, 2 x 75 mL), brine (50 mL), dried with Na_2SO_4 and concentrated in vacuo. The white solid was triturated with ether (using an ultrasound batch) and cooled to 0 °C. The cold suspension was filtered, washed with cold ether (2 x 10 mL0 and dried in vacuo to give **8** (**C31**) as a white powder (1.66 g, 90%). Analytical data were in agreement with data reported in patent WO 2014143799 (see below).

Analytical data for compound 8 (C31): R_f 0.53 (3:2 EtOAc:Pet.Ether); ^{1}H NMR (500 MHz, chloroform-d) δ 7.45 (t, *J* = 8.2 Hz, 1H), 7.40 (d, *J* = 8.4 Hz, 2H), 7.32 (d, *J* = 8.5 Hz, 2H), 7.25 – 7.20 (m, 2H), 7.16 (d, *J* = 8.3 Hz, 1H), 5.42 (s, 2H), 4.19 (t, *J* = 6.1 Hz, 2H), 3.53 (t, *J* = 5.5 Hz, 2H), 3.48 (br s, 1H), 3.45 (s, 3H), 1.94 – 1.87 (m, 2H); ^{13}C NMR (126 MHz, chloroform-d) δ 155.39 (Cq), 153.64 (Cq), 152.94 (Cq), 151.71 (Cq), 149.85 (Cq, q, *J* = 2.0 Hz), 146.38 (Cq), 134.66 (Cq), 134.25 (Cq), 130.75 (CH) 129.90 (2×CH), 129.29 (2×CH), 120.46 (CF_3, q, *J* = 258.4 Hz), 118.37 (CH), 118.00 (CH), 113.13 (CH), 103.09 (Cq), 58.68 (CH_2), 46.76 (CH_2), 37.89 (CH_2), 31.02 (CH_2), 30.08 (CH_3); ^{19}F NMR (282 MHz, chloroform-d) δ -57.95 (CF_3); HRMS (ESI): calcd. For $C_{23}H_{21}ClF_3N_4O_5^+$ $[M+H]^+$ 525.1147, found 525.1148.

Figure S2. ^{1}H NMR spectrum of C31 batch 1 in CD_3OD

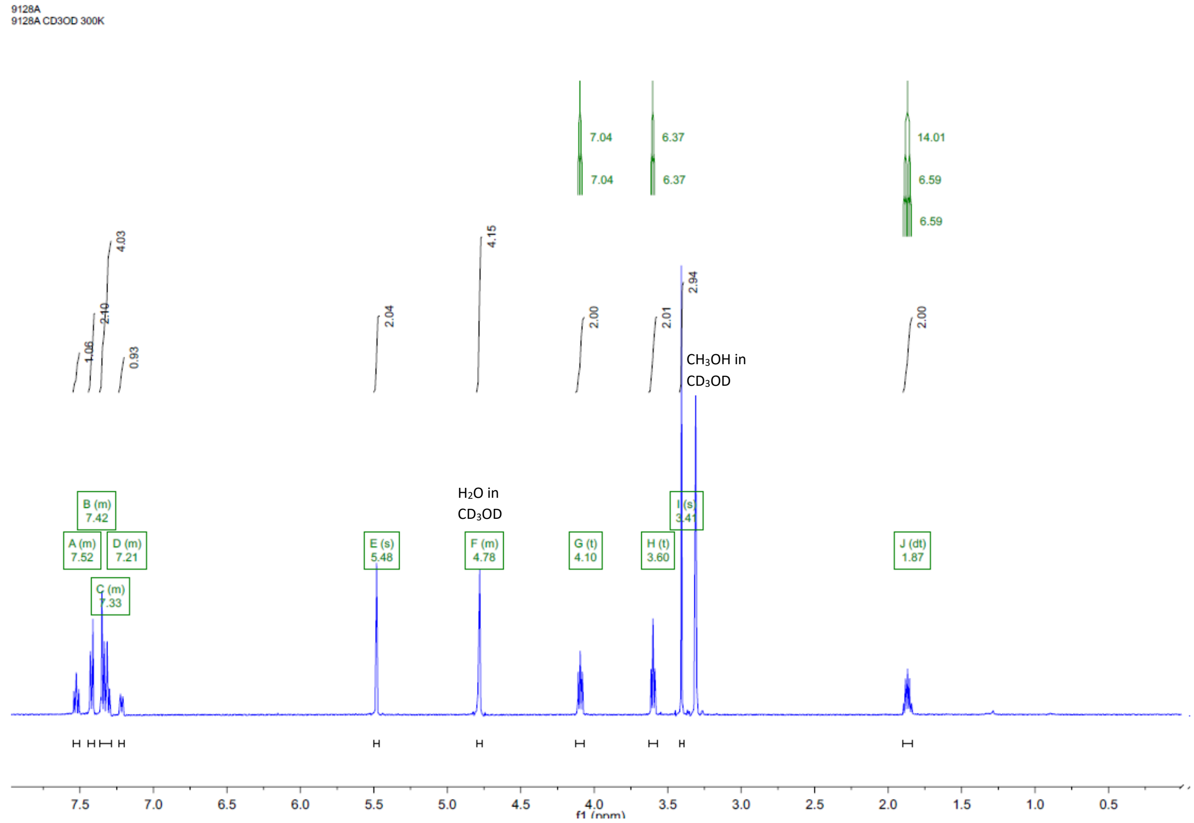

Figure S3. ^{1}H NMR spectrum of C31 batch 2 in chloroform-*d*

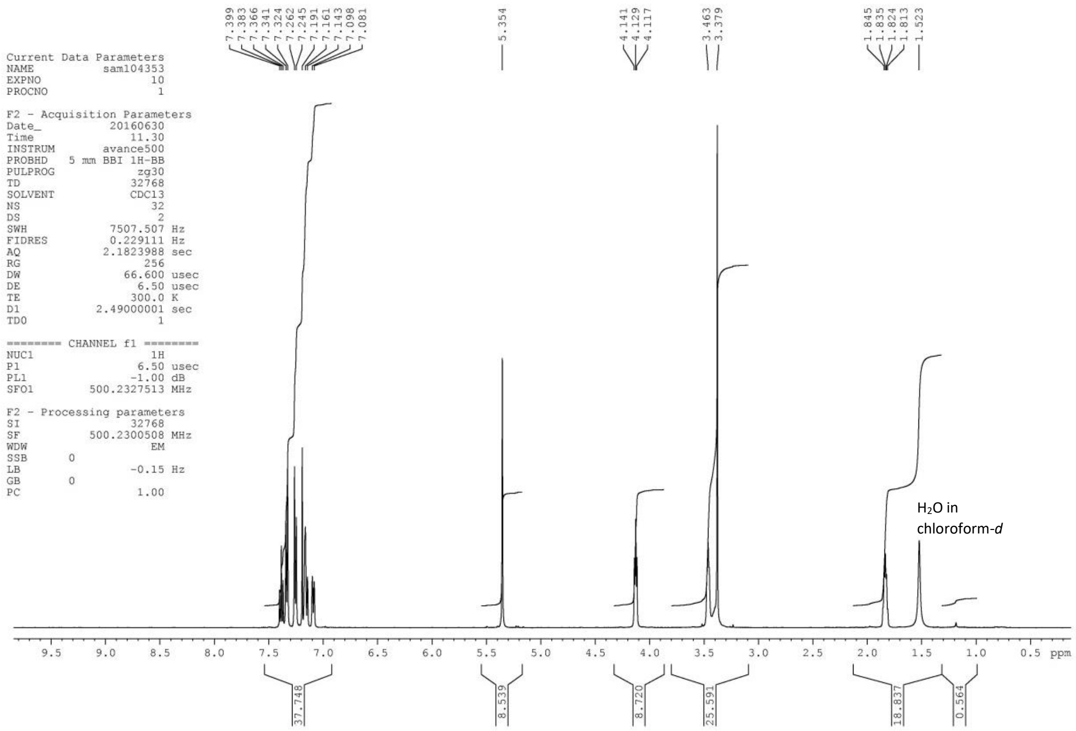

Figure S4. ^{1}H NMR, ^{13}C, DEPT135 and ^{19}F spectra of C31 batch 3 in chloroform-*d*

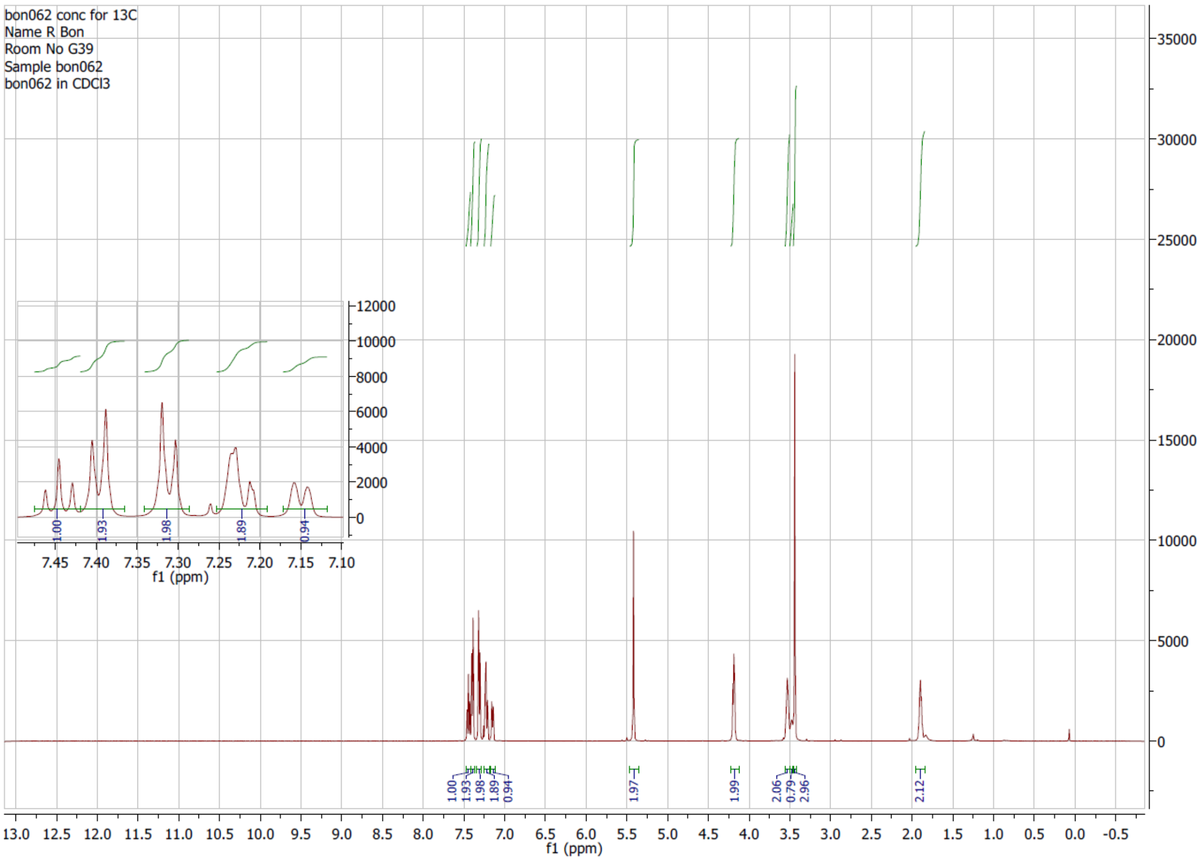

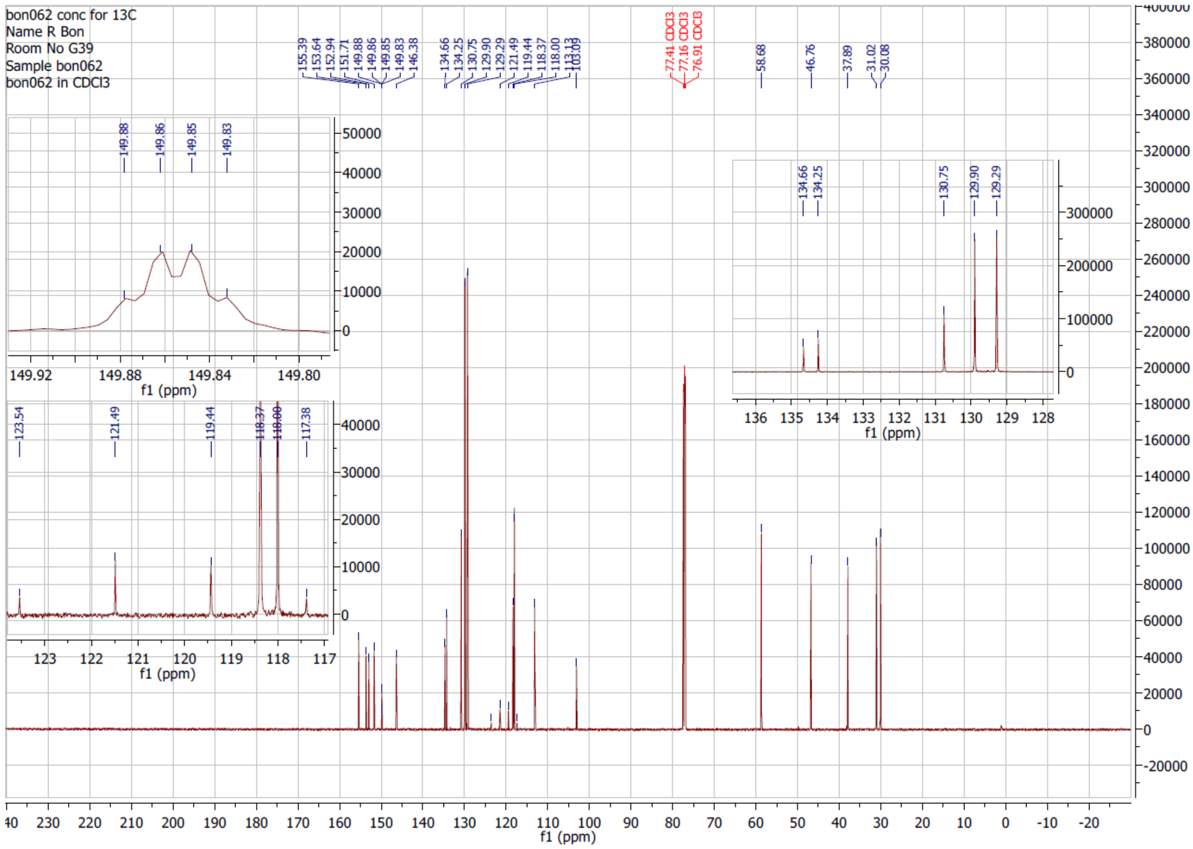

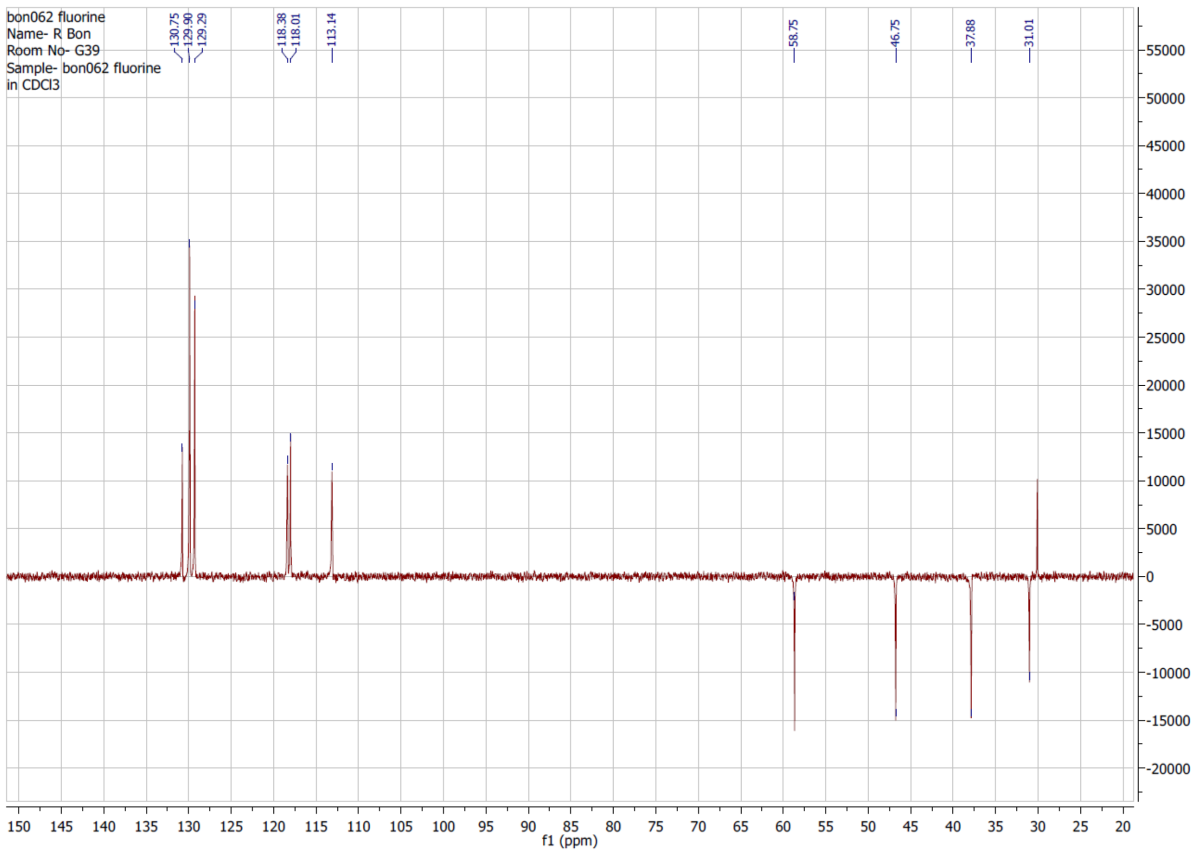
bon062 fluorine
Name- R Bon
Room No- G39
Sample- bon062 fluorine
in CDCl3
130.75
129.90
129.29
118.38
118.01
113.14
58.75
46.75
37.88
31.01
f1 (ppm)

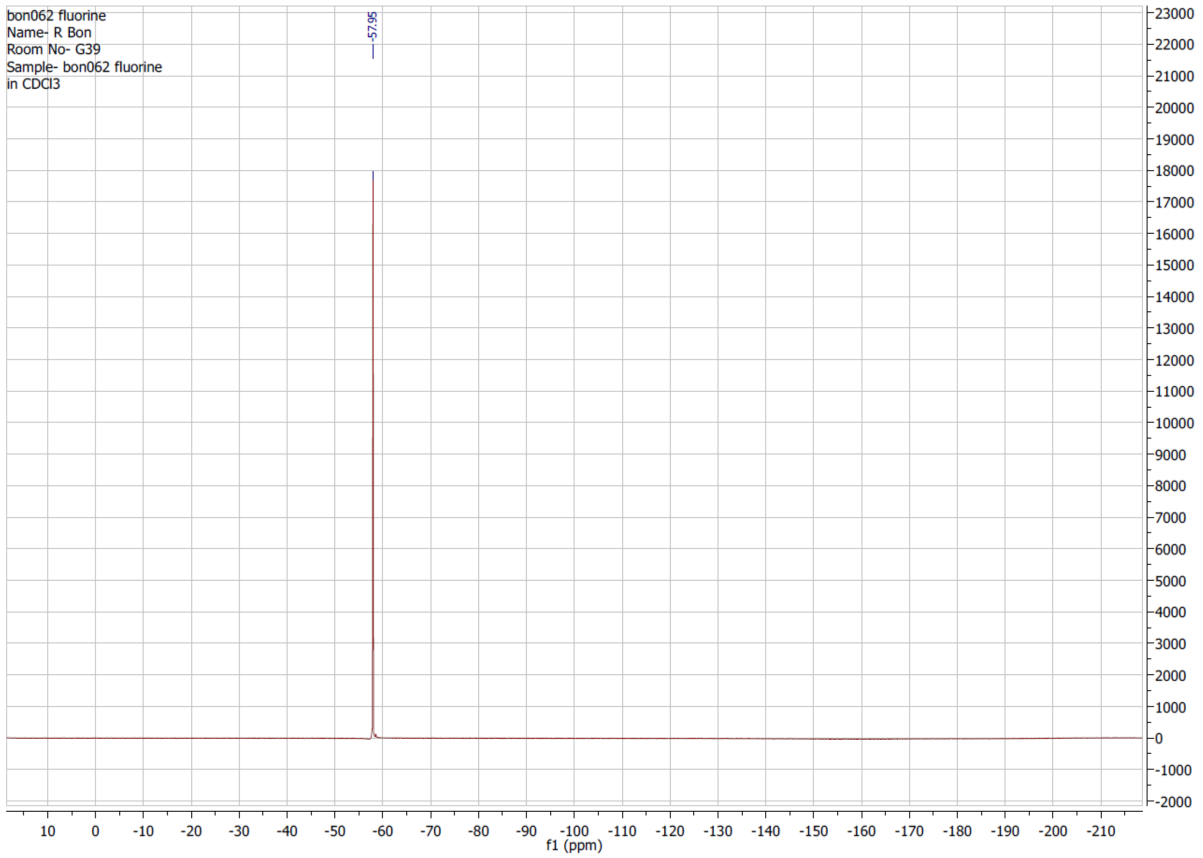
bon062 fluorine
Name- R Bon
Room No- G39
Sample- bon062 fluorine
in CDCl3
-57.95
f1 (ppm)

Comparison of three different batches of C31

^{1}H NMR showed that all three batches of **C31** were analytically pure (Fig S2–S4). Stock solutions of the three different batches of **C31** (10 mM in DMSO) were prepared by two different investigators. The concentrations of these DMSO stock solutions were compared by ESI-LC-MS. For this, DMSO stock solutions were diluted to 1 mM with DMSO. All three batches were then analysed by calcium recording in HEK293 cells conditionally expressing TRPC4-C1 concatemeric channels. Data are shown below.

Figure S5. ESI-LC-MS spectrum for 10 mM DMSO stock solution 1 (diluted 1:10)

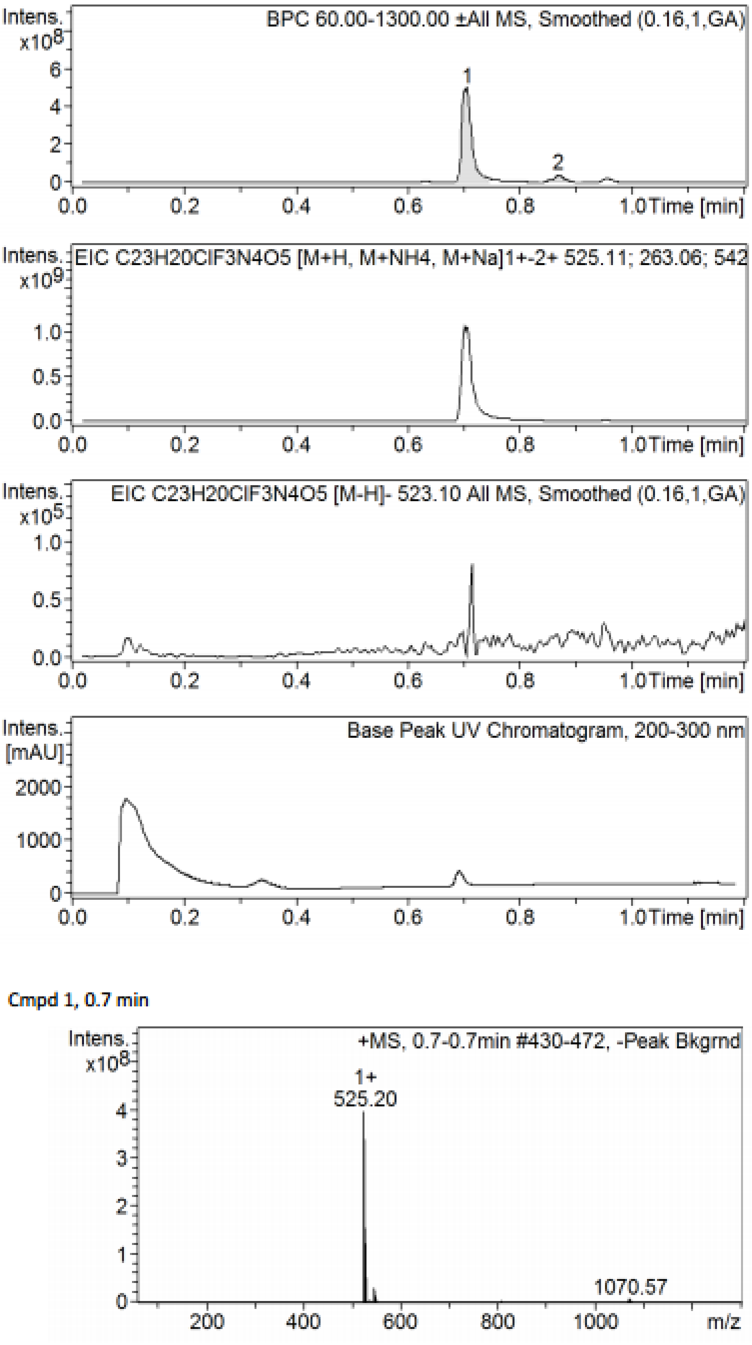

Figure S6. ESI-LC-MS spectrum for 10 mM DMSO stock solution 2 (diluted 1:10)

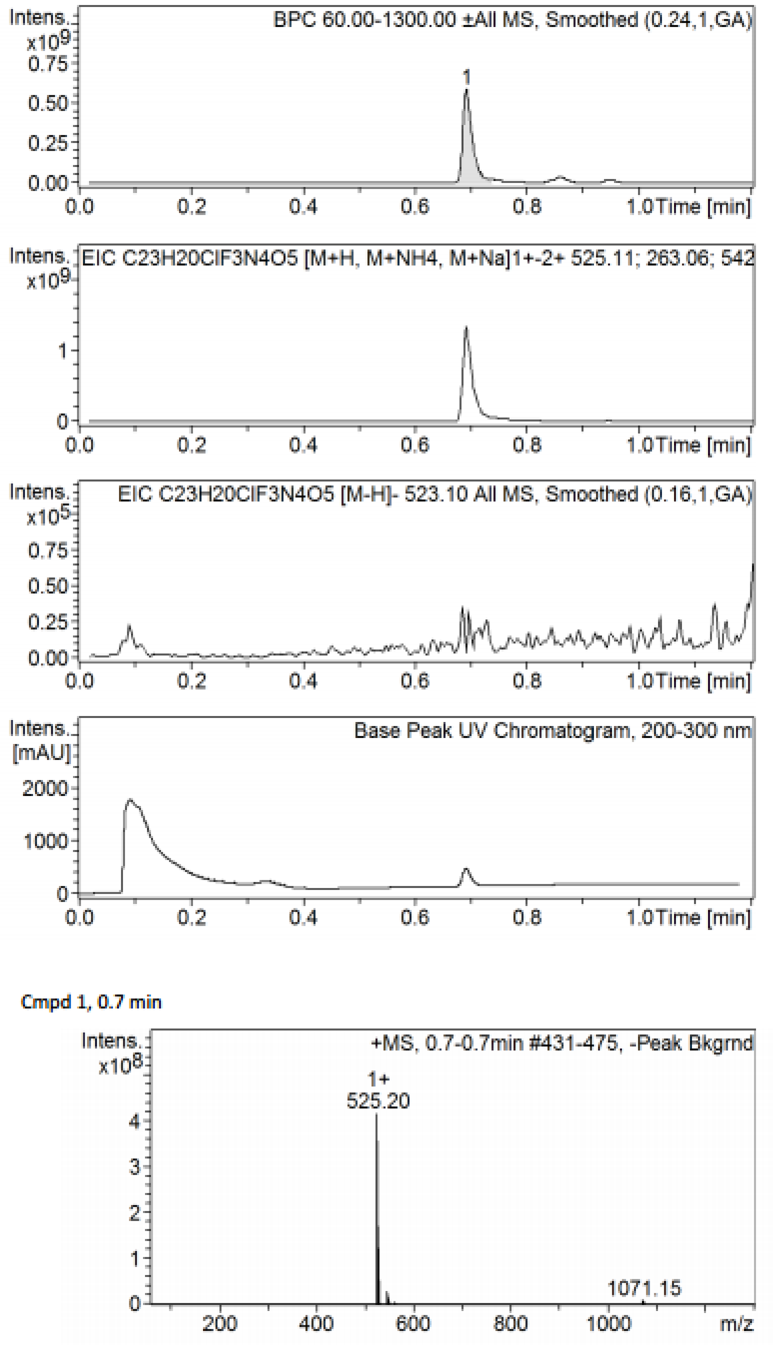

Figure S7. ESI-LC-MS spectrum for 10 mM DMSO stock solution 3 (diluted 1:10)

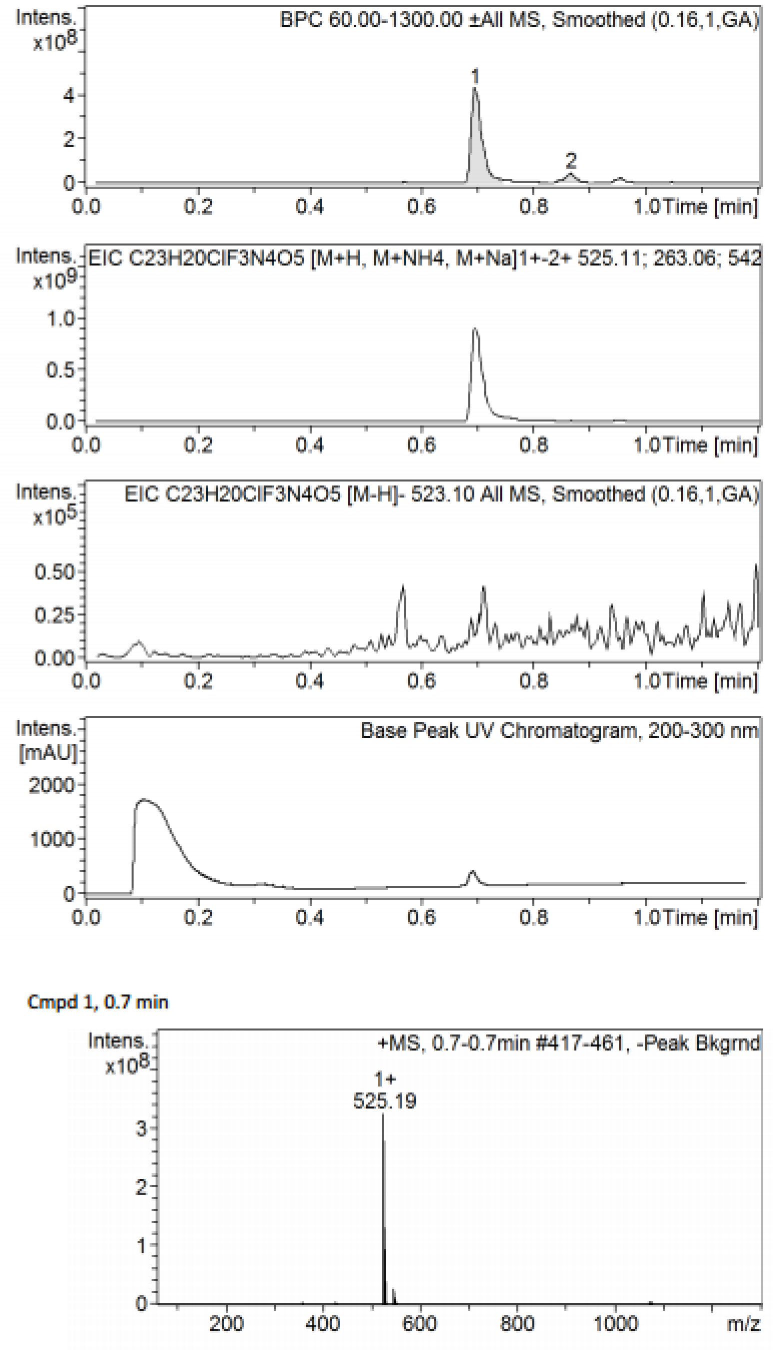

The ESI-LC-MS data in Figures S5-S7 show that the three different 10 mM DMSO stock solutions 1-3 (made from batches 1-3 of C31) were of equal purity and concentration.

Figure S8. Fluorometric recordings (ΔF; with fura-2 as ratiometric, intracellular Ca^{2+} indicator and 10 nM (-)EA as agonist; n/N = 6/18) of three batches of C31 at 1 nM.

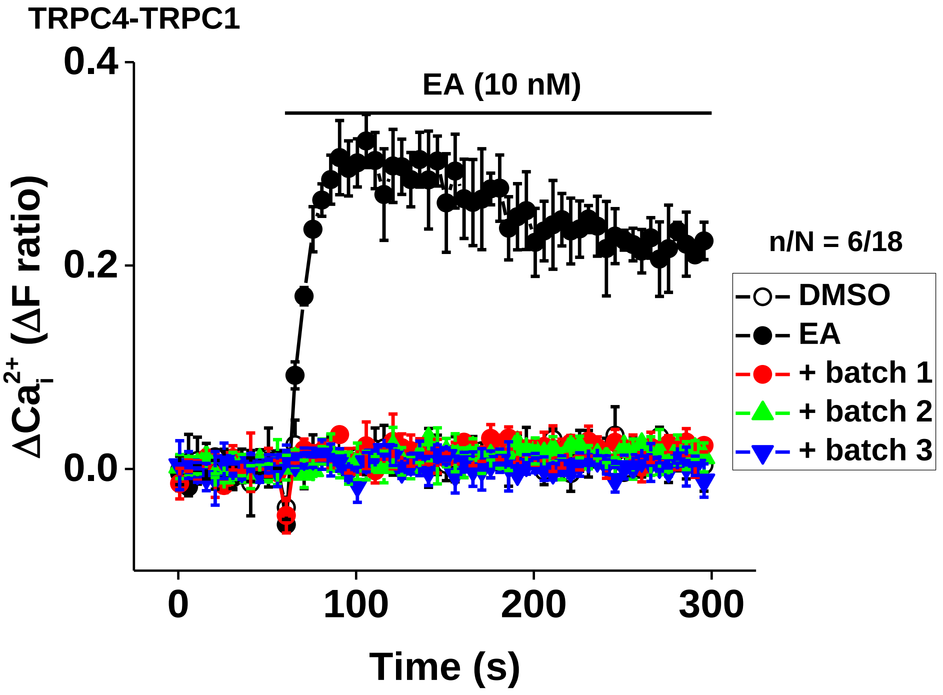

All three batches of **C31** fully inhibited (-)-Englerin A-activated TRPC4-TRPC1 concatemeric channels at a concentration of 1 nM.
